# Multi-omics-based prediction of hybrid performance in canola

**DOI:** 10.1007/s00122-020-03759-x

**Published:** 2021-02-01

**Authors:** Dominic Knoch, Christian R. Werner, Rhonda C. Meyer, David Riewe, Amine Abbadi, Sophie Lücke, Rod J. Snowdon, Thomas Altmann

**Affiliations:** 1 Department of Molecular Genetics, Leibniz Institute of Plant Genetics and Crop Plant Research (IPK), 06466 Seeland, OT Gatersleben Germany; 2 The Roslin Institute, University of Edinburgh, Easter Bush, Midlothian, EH25 9RG Scotland, UK; 3 Institute for Ecological Chemistry, Plant Analysis and Stored Product Protection, Julius Kühn Institute (JKI)—Federal Research Centre for Cultivated Plants, 14195 Berlin, Germany; 4 NPZ Innovation GmbH, Hohenlieth, 24363 Holtsee, Germany; 5 Norddeutsche Pflanzenzucht Hans-Georg Lembke KG, Hohenlieth, 24363 Holtsee, Germany; 6 Department of Plant Breeding, IFZ Research Centre for Biosystems, Land Use and Nutrition, Justus Liebig University, Heinrich-Buff-Ring 26-32, 35392 Giessen, Germany

**Keywords:** Spring-type *brassica napus* (canola), Hybrid prediction, Genomic best linear unbiased prediction (gBLUP), Reproducing kernel Hilbert space regression (RKHS), Heterosis, Agronomic traits

## Abstract

**Complementing or replacing genetic markers with transcriptomic data and use of reproducing kernel Hilbert space regression based on Gaussian kernels increases hybrid prediction accuracies for complex agronomic traits in canola**.

In plant breeding, hybrids gained particular importance due to heterosis, the superior performance of offspring compared to their inbred parents. Since the development of new top performing hybrids requires labour-intensive and costly breeding programmes, including testing of large numbers of experimental hybrids, the prediction of hybrid performance is of utmost interest to plant breeders. In this study, we tested the effectiveness of hybrid prediction models in spring-type oilseed rape (*Brassica napus* L./canola) employing different omics profiles, individually and in combination. To this end, a population of 950 F_1_ hybrids was evaluated for seed yield and six other agronomically relevant traits in commercial field trials at several locations throughout Europe. A subset of these hybrids was also evaluated in a climatized glasshouse regarding early biomass production. For each of the 477 parental rapeseed lines, 13,201 single nucleotide polymorphisms (SNPs), 154 primary metabolites, and 19,479 transcripts were determined and used as predictive variables. Both, SNP markers and transcripts, effectively predict hybrid performance using (genomic) best linear unbiased prediction models (gBLUP). Compared to models using pure genetic markers, models incorporating transcriptome data resulted in significantly higher prediction accuracies for five out of seven agronomic traits, indicating that transcripts carry important information beyond genomic data. Notably, reproducing kernel Hilbert space regression based on Gaussian kernels significantly exceeded the predictive abilities of gBLUP models for six of the seven agronomic traits, demonstrating its potential for implementation in future canola breeding programmes.

**Supplementary Information:**

The online version contains supplementary material available at 10.1007/s00122-020-03759-x.

## Introduction

Hybrid varieties are key to crop improvement and future plant breeding strategies due to their outstanding agronomic features, a biological phenomenon known as heterosis. Nowadays, commercial varieties of oilseed rape grown worldwide are predominantly hybrids (Stahl et al. 2017; Liu et al. 2018a). These varieties perform better than open-pollinated varieties, especially under stressful environmental conditions. Hybrid oilseed rape plants can be sown later, due to their early vigour, show higher disease resistance, and have increased vitality and compensation ability, securing high, stable and consistent yields (Qian et al. 2007; Zhang et al. 2017; Liu et al. 2018c). A very important element in implementing hybrid breeding is the recognition of a heterotic pattern that supports high-yielding lines (Zhao et al. 2015). However, in comparison with other important hybrid crops like maize, canola displays relatively low levels of *F*_1_ heterosis (Radoev et al. 2008). This can be attributed to the fact that hybrid breeding in rapeseed began only a few decades ago after suitable hybrid seed production systems were developed, and therefore, large and well-defined heterotic pools have not been established yet (Melchinger and Gumber 1998; Kole 2007). However, several attempts were made to broaden the genetic diversity and to develop heterotic gene pools for rapeseed hybrid breeding (Qian et al. 2007; Girke et al. 2012; Jesske et al. 2013; Li et al. 2014), because the breeding industry has considerable interest in utilisation of heterosis to improve plant performance under optimal and stressful conditions. The identification of new superior hybrids among the millions of possible crosses among new parental lines generated every year requires extensive testing programmes, involving the production of numerous testcrosses, extensive multi-location/-year field trials to generate phenotypic data and to test hybrid performance. As such programmes demand considerable economical and logistical efforts (Desta and Ortiz 2014), the prediction of hybrid performance is highly desirable for breeders. An accurate prediction of the expression of traits with a complex genetic architecture would allow for a straightforward preselection of a few hundred most favourable crosses/hybrids with high success rate, could substantially reduce the volume of the labour-intensive and time-consuming field trials (Xu et al. 2016; Kadam et al. 2016), and would greatly impact the efficiency of hybrid breeding (Longin et al. 2015).

Whole-genome prediction constitutes a powerful tool that revolutionized plant breeding (Crossa et al. 2014; Technow et al. 2014; Xu et al. 2014; Heslot et al. 2015; Mangin et al. 2017; Hickey et al. 2017). A commonly used method is genomic best linear unbiased prediction (gBLUP; Meuwissen et al. 2001), which computes the genetic merits from a genomic relationship matrix and has been shown to be equivalent to ridge-regression best linear unbiased prediction (rrBLUP;Whittaker et al. 2000; Habier et al. 2007). However, these approaches are mostly restricted to the incorporation of additive and dominance effects, and often ignore intricate epistatic interactions due to computationally demand (Jiang and Reif 2015). Taking epistasis into account can increase prediction accuracies (Hu et al. 2011; Wang et al. 2012; Muñoz et al. 2014; He et al. 2016). Meuwissen et al. (2001) tried to relax the assumption made by RR-BLUP that genetic effects are evenly spread across the genome (homoscedastic marker variances) using Bayesian models. In addition, kernel-based methods like reproducing kernel Hilbert space regression (RKHS) have been exploited for predictions (Gianola and van Kaam 2008). They allow a great deal of flexibility and make no assumptions of linearity, which may render them superior in their ability to capture non-additive genetic effects. However, there is no universally best prediction model (Momen et al. 2018).

The approaches mentioned above focused on modelling of additive and non-additive effects using matrices based on genetic markers. However, there is evidence that genomic prediction may not be capable to capture all complex gene interactions and downstream regulatory processes, even with complete sequence information available (Zhu et al. 2012; Ritchie et al. 2015). Therefore, the utilisation of endophenotypes such as gene expression or metabolite abundances, which reflect intermediate steps from genotype to phenotype, was proposed to improve the prediction of complex traits, as they are expected to represent more closely the variability across genotypes than genomic data per se (Mackay et al. 2009; Patti et al. 2012; Civelek and Lusis 2014). Previous studies in Arabidopsis (Meyer et al. 2007; Gärtner et al. 2009; Steinfath et al. 2010), maize (Riedelsheimer et al. 2012; Feher et al. 2014), and rice (Dan et al. 2016, 2018; Xu et al. 2016; Wang et al. 2019) have shown that metabolite levels can have high predictive power and can in some cases (Zhao et al. 2015) improve prediction accuracies. Other studies illustrate the predictive value of transcriptome data (Swanson-Wagner et al. 2006; Fu et al. 2012; Zenke-Philippi et al. 2016, 2017), including small RNAs (Seifert et al. 2018). Compared to genomic data, transcripts have the advantage that they are independent of marker LD and are therefore better suited for prediction across heterotic pools (Frisch et al. 2010). Downstream omics data, including expression data and metabolite profiles, are expected to integrate interactions within and between biological layers, thus they may capture physiological epistasis (Westhues et al. 2017). In particular, the integration of endophenotypes with genetic markers has been shown to significantly improve predictive abilities in maize and rice (Guo et al. 2016; Westhues et al. 2017; Schrag et al. 2018; Wang et al. 2019).

The goal of the present study was to evaluate the suitability of omics-based models to predict hybrid performance in spring-type oilseed rape that can be effectively implemented in a commercial breeding programme. To this end, a population of 950 hybrids has been evaluated in field trials, and genetic marker, global transcriptome and polar metabolite data were collected from their respective parental lines. We evaluated the performance of two different models, simple (genomic) best linear unbiased predictions (gBLUP) and reproducing kernel Hilbert space regressions (RKHS) based on a Gaussian kernel for prediction of hybrid performance under field and glasshouse conditions. We focused on the following questions: (1) can hybrid performance in the field be effectively predicted using parental line genotype information, (2) can other parental omics data sets collected in controlled conditions be utilised to effectively predict hybrid performance and how do their prediction accuracies compare with that of genetic markers, (3) can prediction accuracy be increased by stacking multiple omics sets and which combinations are the most promising for which set of traits, (4) can per se line and hybrid performance in the glasshouse be effectively predicted using the omics data sets, and (5) can higher prediction accuracies be achieved for agronomic traits in canola by employing reproducing kernel Hilbert space regression models compared to gBLUP?

## Materials and methods

### Genetic material and generation of an F_1_ hybrid population

The experimental materials consisted of 477 spring-type *B. napus* (canola) genotypes with double-low seed quality (low erucic acid and low glucosinolate content) from a previously described breeding programme (Jan et al. 2016, 2019; Knoch et al. 2020). The largest proportion, 475 lines, comprised genetically diverse pollinator lines that could be assigned to three breeding pools (referred to as breeding pools 1, 2 and 3). The pollinator lines were derived from 184 crosses, with some elite parents used in several crosses. The F_1_ hybrid population with 950 individuals was created by crossing the 475 pollinators with two elite male-sterile testers (MS1 and MS2) from a pool of testers carrying the *Male Sterility Lembke* (MSL) sterility system (NPZ Lembke, Hohenlieth, Germany).

### Reference genome version and genotype data

The 477 parental genotypes were analysed using the *Brassica* Infinium 60k genotyping array (Illumina Inc., San Diego, CA; USA). Genotyping data, including single nucleotide polymorphisms (SNPs) and copy-number variations (CNVs), were generated using the ‘gsrc’ R package (Grandke et al. 2017) and filtered as described in Knoch et al. (2020). The data set is available from the e!DAL—Plant Genomics and Phenomics Research Data Repository (http://dx.doi.org/10.5447/IPK/2019/10). An improved version of the *Brassica napus* cv. Darmor v4.1 reference genome assembly (Chalhoub et al. 2014), generated by integrating long read information (NRGene, DeNovoMAGIC™; unpublished data from David Edwards, University of Western Australia) into the pseudomolecules, was used to position the SNP markers. Missing SNP calls were imputed using the BEAGLE v.4.1 implementation of the ‘synbreed’ R package (Wimmer et al. 2012). A total of 16,311 markers comprising 13,201 unique, single-copy SNPs (Data S1), 3106 deletions and four duplications remained after filtering. Transcripts of the reference genome used were predicted de novo and annotated as described in Knoch et al. (2020).

### Field trials and evaluation of agronomic traits

In 2012, field trials were conducted in an augmented unreplicated design by the commercial partners NPZ Lembke KG (NPZ) and Deutsche Saatveredelung AG (DSV). Hybrids were evaluated at eight different locations in Denmark (Abildgard, Dyngby), Germany (Roßleben, Hohenlieth), Poland (Słupia, Krzyżewo), Latvia (Jelgava) and Estonia (Viljandi), chosen according to the origin of the crosses (adapted European, crosses with exotics, crosses with Australian and winter-spring crosses); thus, each hybrid was represented at least at three locations (Data S1). All hybrids were tested in one common location in Poland (Krzyżewo). Four commercial lines (‘Achat’, ‘Osorno’, ‘Mirakel’ and ‘DLE 1108’) were included as standards at all locations. These standards were analysed as duplicates in all but one trial. Various traits of agronomic importance were evaluated, including the content of total seed glucosinolates (GSL; μmol/g seed), the days to onset of flowering (DTF; measured as number of days from sowing until 50% flowering plants per plot), the seed oil yield (dt/ha), seedling emergence (visual observation ranging from a minimum value of 0 to maximum 10), the seed oil content (% volume per seed dry weight), the seed protein content (% volume per seed dry weight) and seed yield (dt/ha). Best linear unbiased estimators for each agronomic trait (BLUEs, Data S1) were calculated using a linear mixed model (Eq. 1) implemented in R using the ‘lme4’ package (Bates et al. 2015). In the models, $Y$ denotes the phenotypic value of a trait for each genotype, $G$ represents the fixed effect of the Genotype, $T$ the random effect of the Trial, $L$ the random effect of the Location, $T \times L$ the Trial-Location-Interaction, $L \times G$ the Location-Genotype-Interaction, and $e$ the residual error (errors were assumed to be normally, independently and identically distributed). Broad-sense heritabilities (H^2^) for each trait are estimated by Eq. 2, where $\sigma_{G }^{2}$ and $\sigma_{{\text{e}}}^{2}$ denote the variance components of the genotype and the residual variance, and $n_{0 }$ the number of plant replicates (*n* = 4) per genotype (Nakagawa and Schielzeth 2010; He et al. 2016). Variance components $\sigma_{G }^{2}$ and $\sigma_{{\text{e}}}^{2}$ were estimated by restricted maximum likelihood (REML) and extracted from the mixed linear models (Eq. 1) assuming that all effects were random effects. All statistical analyses were performed in the R software environment for statistical computing and graphics version 3.4.2 (R Core Team 2019) and RStudio Version 1.1.419.

1 $$Y = G + T + L + TxL + LxG + e$$

2 $$H^{2} = \frac{{\sigma_{G}^{2} }}{{\sigma_{G}^{2} + \frac{1}{{n_{0} }} \sigma_{{\text{e}}}^{2} }}$$

### Plant cultivation under controlled conditions and high-throughput phenotyping

Plants were cultivated and phenotyped in the IPK phenotyping facility for large plants (Junker et al. 2015) for four weeks with three replicates per genotype as described in Knoch et al. (2020). Each replicate comprised a pot with nine plants. Parental genotypes were analysed in an incomplete randomized block design with four phenotyping experiments in the spring and winter period of the year 2014. An additional experiment was conducted in spring 2015 with a selection of 120 hybrids, composed of 60 high and 60 low performers according to their seed yield in the field in the year 2012. The hybrids were grown under the same climate regime as the parental genotypes. BLUEs for the image-derived phenotypic traits, including projected leaf area and estimated biovolume, were calculated using all five phenotyping experiments. At the end of the experiments, the end-point biomasses (fresh and dry weight) of the plants were determined. Processed phenotype data were taken from Knoch et al. (2020).

### Sampling of early vegetative shoot material and post-processing

The shoot material from four plants per container was collected at 14 days after sowing (DAS), between seven to nine hours after illumination, and immediately shock-frozen in liquid nitrogen. Plant material was homogenised in a scintillation vial (Zinsser Analytic GmbH, Eschborn, Germany) for 2 min at −60 °C using two 8-mm steel balls and a cryogenic plant grinding and dispensing system (Labman Automation Ltd., Stokesley, United Kingdom). The material of four individual plants per carrier was combined and equal amounts of plant material from all carriers from the different experiments were pooled. Samples from the first phenotyping experiment were omitted, as a breakdown of the cooling system in the glasshouse caused elevated temperatures during this experiment and an increased developmental speed, which might have biased the results. 15 ± 1.5 mg fresh weight were dispersed in 1.4 ml storage tubes (Micronic, Lelystad, The Netherlands) for metabolite profiling. For transcript profiling, 50 ± 1.5 mg fresh weight were manually weighed. Material was stored at −80 °C until use.

### Metabolite profiling in early vegetative tissue

Polar metabolites were extracted from 15 mg deep-frozen, homogenised plant material using a previously described liquid–liquid extraction protocol (Lisec et al. 2006; Riewe et al. 2012, 2016). The protocol was adjusted to 96 tubes/rack format and implemented on a liquid handling system (Biomek® FXP, Beckman Coulter GmbH, Krefeld, Germany): 0.625 ml chilled extraction buffer (2.5:1:1 *v/v* MeOH/CHCl_3_/H_2_O) and 0.250 ml H_2_O after incubation. Extraction was split into six batches with 96 samples each and aliquots of 50 μl of polar phase were sampled. The dried extracts were in-line derivatised directly prior to injection according to Erban et al. (2007) using a Gerstel MPS2-XL autosampler (Gerstel, Mühlheim/Ruhr, Germany) and analysed in split mode (1:4) using a LECO Pegasus HT time-of-flight mass spectrometer (LECO, St. Joseph, MI, USA) hyphenated with an Agilent 7890 gas chromatograph (Agilent, Santa Clara, CA, USA) as previously described by Riewe et al. (2012, 2016) and Wiebach et al. (2020). A total of 27 quality control pools (pooled material from all samples), and eight negative controls (extraction procedure with empty vials; ‘blanks’) were included in the analysis for quality control.

### Metabolite data processing and normalisation

Analyte mass spectra were deconvoluted using the LECO ChromaTOF software including the ‘Statistical Compare’ package. Chromatographic peaks were annotated by querying the electron impact spectra library provided by the Golm Metabolome Database (GMD, http://gmd.mpimp-golm.mpg.de). Quantitative peak information was extracted with the R package ‘TargetSearch’ (Cuadros-Inostroza et al. 2009). Data were filtered for contaminations using a sample to blank ratio > 2. In total, 154 analytes of biological origin, 64 of known and 90 of unknown chemical structure, were quantified. The metabolite intensities were normalised regarding to sample weight and measuring day/detector response. Outliers were removed (median ± 4 × SD), and metabolite data were power transformed to ensure an approximate normal distribution (Box and Cox 1964). The complete list of annotated metabolite peaks is provided as Data S1.

### Transcriptome profiling

Total RNA was isolated from each sample using the GeneJET Plant RNA Purification Mini Kit (Thermo Fischer Scientific Inc., Waltham, USA) according to the manufacturer’s protocol and eluted in 50 µl nuclease-free water. RNA quantity and purity were assessed using a NanoDrop One Microvolume UV–Vis Spectrophotometer, and RNA integrity number (RIN) was checked for each fourth RNA sample using a Bioanalyzer 2100. RNA was diluted to 100 ng/µl and 30 µl were used for sequencing in randomized order. cDNA libraries were constructed using the Lexogen SENSE mRNA-Seq Library Prep Kit V2 (Lexogen GmbH, Vienna, Austria). Sequencing was performed using 100 bp single end (SE) reads on a HiSeq 2500 platform (Illumina, San Diego, USA). In total, 27 flow cell lanes on five high-output and rapid-run flow cells were used, resulting in a total of approximately 520 Gbases/4.8 billion single-end reads, covering each genotype with on average approximately 9.5 million reads. More than 96% of all 477 samples could be covered with at least 7 million reads. Adapter-trimmed raw reads were further quality trimmed using the Trimmomatic software v0.36 (Bolger et al. 2014) with the following options: SE, HEADCROP:6, LEADING:20, TRAILING:20, SLIDINGWINDOW:4:15, and MINLEN:50. Trimmed high-quality sequences for each line were concatenated and aligned to the Darmor-*bzh* NRGene reference assembly (fasta) file using Hisat2 v2.0.4 (Kim et al. 2015) using default settings, resulting in an overall mean alignment rate of 82.2% with a mean of 67.3% uniquely aligned reads. The enhanced *Brassica napus* cv. Darmor‐*bzh* reference genome assembly and de novo gene annotations were described in Knoch et al. (2020). Counting of features was performed using HTSeq software v0.6.1p1 (Anders et al. 2015) and the NRGene annotation (.gff3) file with information about 126,667 annotated transcripts and the following settings: -t exon, -i Parent, and -s no. Subsequently, raw counts were normalised for sequencing depths and transcript length using the ‘tpm’ procedure (Wagner et al. 2012) and R statistical software. Data were filtered for transcripts with a relevant expression level by applying a cut-off: median tpm ≥ 5 across all samples (Data S1).

### Genomic and omics-based prediction models

Two types of prediction methods were employed: first, (genomic) best linear unbiased prediction (gBLUP; Meuwissen et al. 2001), using relationship matrices and second, reproducing kernel Hilbert space regression (RKHS; Gianola and van Kaam 2008) based on a Gaussian kernel, a nonlinear regression model which can capture both the additive and non-additive effects using distance matrices. Parental lines were ‘crossed’ in silico by combining the two respective parental matrices to extrapolate hybrid profiles (Werner et al. 2017). The resulting matrices had the dimension ‘number of hybrids’ (*n* = 950) times ‘number of features’ (*n*_G_ = 13,201, *n*_T_ = 19,479, *n*_M_ = 154). The columns of these predictor matrices were centred and standardised to result in values between 0 and 2 (G) and 0 and 1 (T and M), respectively. The in silico generated hybrid profiles were used to calculate the realised relationship matrices (VanRaden 2008; Endelman and Jannink 2012).

The gBLUP as well as the RKHS models were implemented using R (R Core Team 2019) and the mmer2 function from ‘sommer’ package (Covarrubias-Pazaran 2016) to solve the mixed model equations. The general gBLUP model is defined by Eq. 3 in which $y$ is an *n* × 1 vector of phenotypic values (BLUEs), *n* the number of hybrids, *μ* a vector of fixed effects that represent the overall mean. In the model $g_{\alpha }$, $g_{\beta }$ and $g_{\gamma }$ are *n* × 1 vectors of random effects, and $Z_{\alpha }$, $Z_{\beta }\, {\text{and}} Z_{\gamma }$ are the design matrices assigning genetic values to hybrids using markers ($g_{\alpha }$), transcripts ($g_{\beta }$), and metabolites ($g_{\gamma }$), respectively. Marker-based genetic values, transcriptomic values and metabolic values of the hybrids were modelled as random effects with $g_{\alpha } \sim N\left( {0, G_{\alpha } \sigma_{\alpha }^{2} } \right)$, $g_{\beta } \sim N\left( {0, G_{\beta } \sigma_{\beta }^{2} } \right)$ and $g_{\gamma } \sim N\left( {0, G_{\gamma } \sigma_{\gamma }^{2} } \right)$, respectively. The term $\sigma_{\alpha }^{2}$ denotes the genomic variance estimated using SNP markers, $\sigma_{\beta }^{2}$ the transcriptomic variance and $\sigma_{\gamma }^{2}$ the metabolic variance and $G_{\alpha }$, $G_{\beta }$ and $G_{\gamma }$ were the realised additive relationship matrices calculated by the A.mat function of the ‘sommer’ R package. The residuals $e$ follow a normal distribution $e \sim N\left( {0, I\sigma^{2} } \right)$, where $I$ is the identity matrix.

We extended our gBLUP models by including the dominance relationship and the second-order additive × additive epistatic relationship matrix based on the SNP markers. The dominance relationship matrix was calculated using the D.mat function implemented in the ‘sommer’ R package (method by Su et al. 2012). The realized epistatic relationship matrix was calculated as the Hadamard product of the additive relationship matrix using the E.mat function of the same R package. Both matrices were used equivalently to the additive genomic relationship matrix.

Analogous to the gBLUP approach, the same general statistical model was used for reproducing kernel Hilbert space regression (RKHS) defined by (Eq. 3). Here $g_{\alpha } \sim N\left( {0, K_{\alpha } \sigma_{\alpha }^{2} } \right)$, $g_{\beta } \sim N\left( {0, K_{\beta } \sigma_{\beta }^{2} } \right)$ and $g_{\gamma } \sim N\left( {0, K_{\gamma } \sigma_{\gamma }^{2} } \right)$ are random effects measured by the genetic markers, transcriptome and metabolome data, respectively. $K_{\alpha }$, $K_{\beta }$ and $K_{\gamma }$ denote the Gaussian Kernels based on SNP, transcriptomic and metabolic markers, respectively. For the RKHS regression, in silico generated hybrid profiles were first transformed into Euclidean distance matrices between individuals based on the respective marker types. Gaussian Kernels were subsequently calculated using these distance matrices and a bandwidth parameter *h* using the R package ‘AlphaMME’ (https://bitbucket.org/hickeyjohnteam/ alphamme). The corresponding bandwidth parameters were estimated from the respective log-likelihood profile generated using the kin.blup function of the ‘rrBLUP’ R package (Endelman 2011).

3 $$y = 1{\upmu } + { }Z_{\alpha } g_{\alpha } + Z_{\beta } g_{\beta } + Z_{\gamma } g_{\gamma } + e$$

Predictions were performed for the seven agronomic traits (BLUEs) of the 950 hybrids, as well as for growth-related traits and biomass of the 120 hybrids, which were phenotyped in the glasshouse experiment. Three sets of predictors (G = genomic, T = transcriptomic and M = metabolic data) were generated for the parental lines (475 pollinators and two male-sterile testers). Predictions were performed for each set of predictors (G, T, M) separately, and in combinations of two or three (GT, GM, TM; GTM). A cross-validation (cv) scheme with 1000 cv-cycles was applied, separating the data in a training set (75%) and a validation set (25%). The phenotypes (BLUEs) of each of the 1,000 validation sets, always containing both hybrids of randomly selected pollinators (e.g. Pol1 × MS1 and Pol1 × MS2), were masked and predicted. Prediction accuracies were obtained as average Pearson product-moment correlation coefficients between predicted (*ŷ*) and observed phenotypes (*y*). Differences in prediction accuracy between the data sets and combinations were analysed using an analysis of variance (ANOVA), followed by a post hoc Tukey test implemented using the R packages ‘stats’ and ‘agricolae’.

As the number of omics features between metabolites, transcripts and genetic markers differed largely, cross-validations were performed and 100 random subsets (*n* = 154 and *n* = 10,000) of the genetic and transcriptomic markers were sampled, respectively. These reduced predictor sets were subjected to the same procedures as described above to compare the predictive abilities of the metabolic features to genetic and transcriptomic markers based on the same number of features for each data set. In addition, the predictive abilities of copy number variation marker (*n* = 3110) data and time-resolved image-derived paternal phenotype data (*n*_P_ = 2,436; obtained over 21 days; for details see: Knoch et al. 2020; Knoch 2020) were evaluated. Predictions of paternal biomass (fresh and dry weight at 29 DAS) was performed using the parental feature matrices instead of the in silico generated hybrid profiles using the same procedures as described above.

### Hybrid performance and heterosis

Mid-parent heterosis (MPH in %) was calculated as difference between hybrid performance (F_1_) and the mean value of the two parents [MP = (P1 + P2)/2] for end-point biomass, projected leaf area, estimated biovolume, and early plant height at all time points with available data (Eq. 4). Best-parent heterosis (BPH in %) was calculated as difference between hybrid performance (F_1_) and the better performing parent (Eq. 5).

4 $${\text{MPH}} = \frac{{\left( {F_{1} - {\text{MP}}} \right)}}{{{\text{MP}}}} \times 100$$

5 $${\text{BPH}} = \frac{{\left( {F_{1} - {\text{BP}}} \right)}}{{{\text{BP}}}}{ } \times 100$$

### Dimensionality reduction and data visualization

The set of 477 canola genotypes was visualised using t-distributed stochastic neighbour embedding (t-SNE), a method for constructing a low-dimensional embedding of high-dimensional data.

The analysis was performed using the panel of 13,201 SNP and 3110 CNV markers and a Barnes-Hut implementation of t-SNE (Rtsne function) provided by the ‘Rtsne’ R package. The following parameters were used: dims = 2, perplexity = 50, theta = 0, pca = T, and max_iter = 1000. Principal component analyses (PCA) were performed for metabolites and transcripts on centred and scaled data using the pca function of the ‘pcaMethods’ R package (Stacklies et al. 2007).

## Results

### Field experiments and statistical evaluation of agronomic traits

**Fig. 1:**
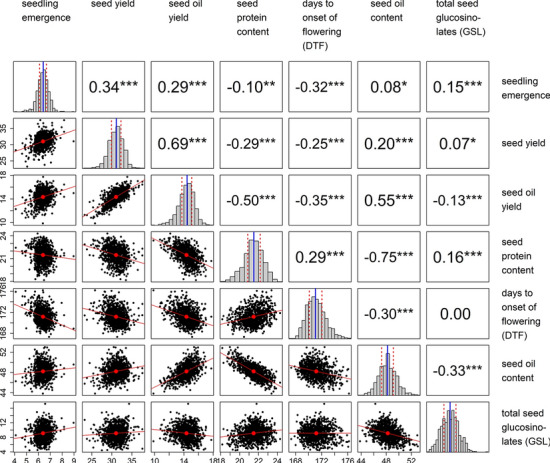
Variation and correlation of agronomic traits analysed in the field trials. Analysis was applied on best linear unbiased estimators (BLUEs) of the seven traits. The lower triangle displays the corresponding bivariate scatter plots. The red dot and the red line correspond to the ellipse centre point and the linear regression fit. The upper triangle displays the Pearson correlation coefficients and significance of the correlations (alpha: * = 0.05; ** = 0.01 and *** = 0.001). The diagonal bar plots display the histograms of the trait distribution. The blue solid line and the dashed red lines correspond to the median and the first and third quantile of the data distribution, respectively

Field trials of 950 F_1_-hybrid genotypes were performed at plant breeding testing sites during the growing season of 2012. Best linear unbiased estimators (BLUEs) were calculated, as in particular the raw data for seed oil yield, DTF and seedling emergence displayed substantial differences between the locations. The BLUEs of all seven traits followed a proximate normal distribution (Fig. 1), but due to missing data BLUEs could be calculated only for 929 of the 950 hybrids. The hybrids displayed substantial phenotypic variation with coefficients of variation ranging from 0.84% for DTF to 20.82% for total seed GSL content (Table 1). In particular, seed yield and seed oil yield were highly positively correlated (*r* = 0.69), while seed oil content and seed protein content displayed a strong negative correlation (*r* = −0.75). Broad sense heritability values (*H*^2^) estimated across the different trials/field locations ranged from 0.34 for the trait seedling emergence to 0.92 for total seed GSL content.

**Table 1:** Summary statistics for agronomic traits evaluated in the field

Trait #	Minimum	1st quartile	Median	Mean	3rd quartile	Maximum	CV (%)	H^2^ (%)
Days to onset of flowering (DTF)	167.34	170.11	171.05	171.18	172.05	176.20	0.84	85
Seedling emergence (good = 9)	4.10	6.10	6.41	6.39	6.68	8.96	7.33	34
Seed GSL (µmol/g)	4.43	7.77	9.13	9.15	10.42	17.23	20.82	92
Seed oil yield (dt/ha)	9.85	13.72	14.44	14.34	15.07	17.74	7.47	82
Seed oil content (%)	43.81	47.25	48.20	48.22	49.13	53.03	2.92	90
Seed protein (%)	18.17	20.86	21.51	21.48	22.18	24.30	4.72	82
Seed yield (dt/ha)	23.22	29.92	31.13	31.04	32.29	37.64	5.89	62

^a^Best linear unbiased estimators (BLUEs) were calculated across the field trials conducted at eight different locations across Europe in 2012

### Description and quality control of the genomic, transcriptomic and metabolic predictor data sets

**Fig. 2:**
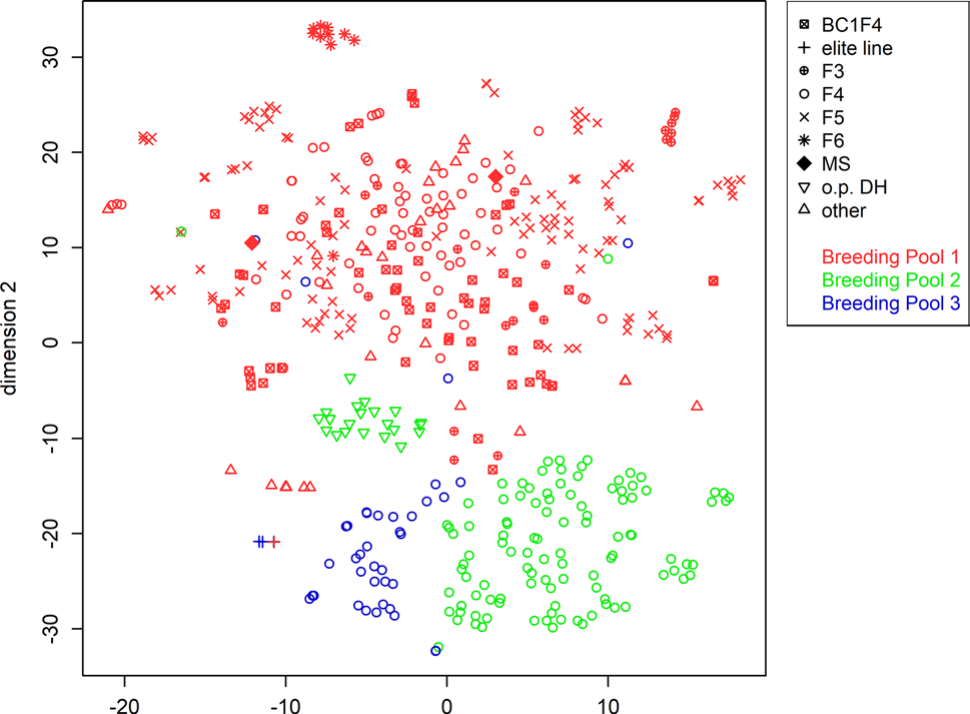
Visualisation of genotypes by t-distributed stochastic neighbour embedding. A Barnes-Hut Implementation of t-distributed stochastic neighbour embedding (t-SNE) was performed on 477 canola genotypes using a panel of 13,201 SNP and 3110 CNV markers. Sample colours indicate assignment of the genotypes to the three breeding pools. Plotting symbols correspond to the population types as indicated in the legend: ‘MS’ = male-sterile mother line and ‘o.p. DH’ = open-pollinated doubled haploid

The 477 parental lines were previously genotyped (Jan et al. 2016; Knoch et al. 2020), yielding a total of 13,201 filtered unique, single-copy SNP and 3110 CNV markers. The population displays a high genetic diversity as visualized by t-distributed stochastic neighbour embedding based on the genetic markers (t-SNE; Fig. 2). The three known breeding pools and subgroups of the breeding material (e.g. F6, open-pollinated DH or elite lines) can be discriminated. Array-derived genotype data for the parental lines was complemented by extensive omics data sets. In the RNA-Seq profiles, 54,521 transcripts (43% of all 126,667 de novo annotated transcripts) were detected as expressed (median tpm > 0) in the sampled shoot material. The subset of 19,479 transcripts (15.38%) quantified at a median level ≥ 5 tpm across all samples was used for subsequent analyses. An explorative principal component analysis (PCA) of the transcriptome data indicated a clustering of genotypes in the fourth principal component (explaining 3.1% of the total variance) that corresponds to the breeding pools underlying the population investigated (Figure S1 a). Global metabolite profiles with relative quantities of 154 metabolites, 64 of known and 90 of unknown chemical structure were also subjected to a PCA, which partially separated the breeding pools in the third PC (Figure S1 b). As expected, quality control pools (consisting of equal amounts of all samples), which had been included to assess the stability of measurements across the long GC–MS analysis, cluster in the centre of the PCA plot.

### Prediction of hybrid performance in the field using individual and combined data sets

**Fig. 3:**
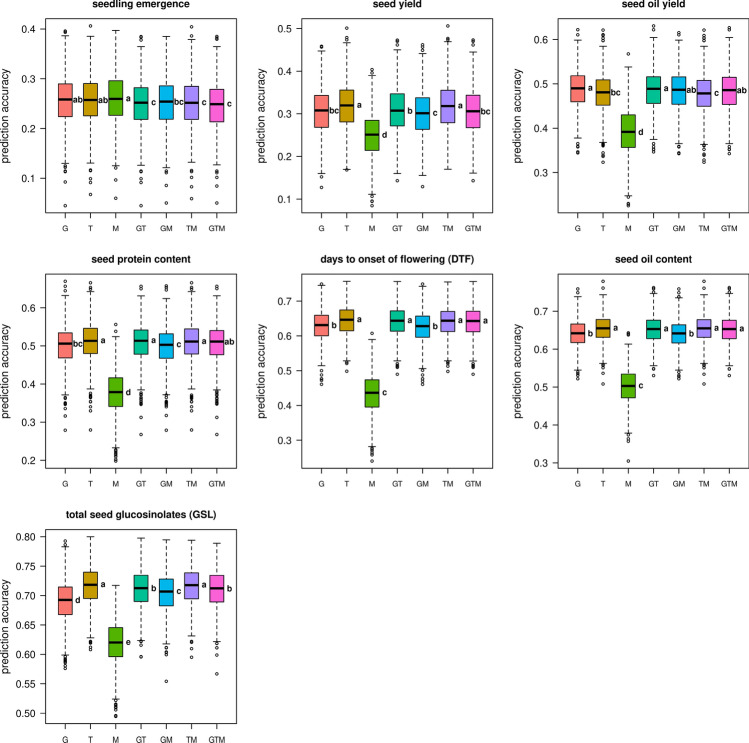
Prediction of hybrid field performance by (genomic) best linear unbiased prediction models. A summary of (genomic) best linear unbiased predictions (gBLUP) of hybrid performance using additive relationship matrices is given as boxplots. Seven agronomic traits, assessed in multi-location field trials, were analysed. The different omics data sets (predictors) were obtained from the parental lines and are denoted as: G = SNP-based genotype data, T = transcriptomic data, and M = metabolite data and their respective combinations GT, GM, MT and GTM. The T and M data sets were obtained from plants cultivated in the glasshouse. Letters beside the boxes indicate significant differences between predictor sets determined by a one-way ANOVA followed by a post hoc Tukey’s multiple comparison test

Using the best linear unbiased estimators (BLUEs) of all seven agronomic traits, (genomic) best linear unbiased predictions (gBLUP) were performed with omics data sets, comprising SNP markers (*n* = 13,201), transcripts (*n* = 19,479) and metabolites (*n* = 154). These data sets were used individually and in all possible combinations for prediction analyses; prediction accuracies are illustrated in Fig. 3. Across all models and traits, mean prediction accuracies ranged from 0.247 for seedling emergence using all available data sets to 0.717 for total seed GSL content using transcriptome data only. In all cases, prediction accuracies strongly depended on and were proportional to the heritability of the traits (Fig. 3). Only minor differences in prediction accuracy between data sets/combinations were determined for the trait seedling emergence, which displayed the lowest mean prediction accuracies (0.247–0.260) of all traits. For the other six traits, the prediction models solely based on metabolite data showed significantly lower prediction accuracies compared to the other sets of predictors. The trait seed oil yield could most effectively be predicted using the genetic markers and the addition of transcripts and/or metabolites as predictive variables led to no significant change in prediction accuracies. For seed yield, highest prediction accuracies (0.319) could be observed using the transcriptome data set, which was significantly higher than using solely genetic markers (0.308). Similarly, also for the traits seed protein content, days to onset of flowering, seed oil content and total seed GSL content, the transcriptome data set and/or models including transcriptome data displayed higher prediction accuracies than the models using pure SNP data. For the trait total seed GSL content, SNP markers yielded significantly lower prediction accuracies compared to all other data sets and combination, with the exception of the model using metabolites only. Thus, for five out of seven agronomic traits a significant increase in prediction accuracies was achieved by using or adding transcriptome data to the predictive models. Notably, when performing a cross-validation, limiting the number of features for both genetic markers and transcripts to *n* = 10,000, the transcripts still yielded higher prediction accuracies than the SNP markers. However, in contrast to the initial hypothesis, no significant increase in prediction accuracies could be achieved by stacking data of all three omics data sets.

Combining SNP and CNV markers resulted in a significant improvement of prediction accuracies using gBLUP models for three traits: DTF, GSL, and in particular seed yield, increasing the mean prediction accuracy by 1.3% compared to SNP markers only. However, the beneficial effect of adding CNVs was negligible in the stacked models including genetic, transcriptomic, and metabolite data (Data S2).

Dominance and epistasis are known to be an important source of non-additive genetic variance for many agronomic traits. Hence, we explored the effect of a dominance and a second order additive × additive epistatic genomic relationship matrix in our gBLUP models for prediction of agronomic traits.

Combining dominance and/or epistatic relationship matrices, calculated based on SNP markers, with the additive genomic relationship matrix significantly increased the prediction accuracies for five traits (Figure S2; Data S2). The highest increase in median prediction accuracy was observed for seed protein content (2.1%), seed oil yield (3.7%) and seed oil content (4.2%). The specific combination yielding the highest prediction accuracy differed between traits. A detailed comparison of the performance of the individual matrices and combinations is given in Data S2. Stacking of the additive, dominance and epistatic genomic relationship matrices with transcript and metabolite data did further improve prediction accuracies for seed protein content, DTF, seed oil content, and GSL (Data S2).

### Comparison of the predictive abilities of gBLUP and RKHS models

**Fig. 4:**
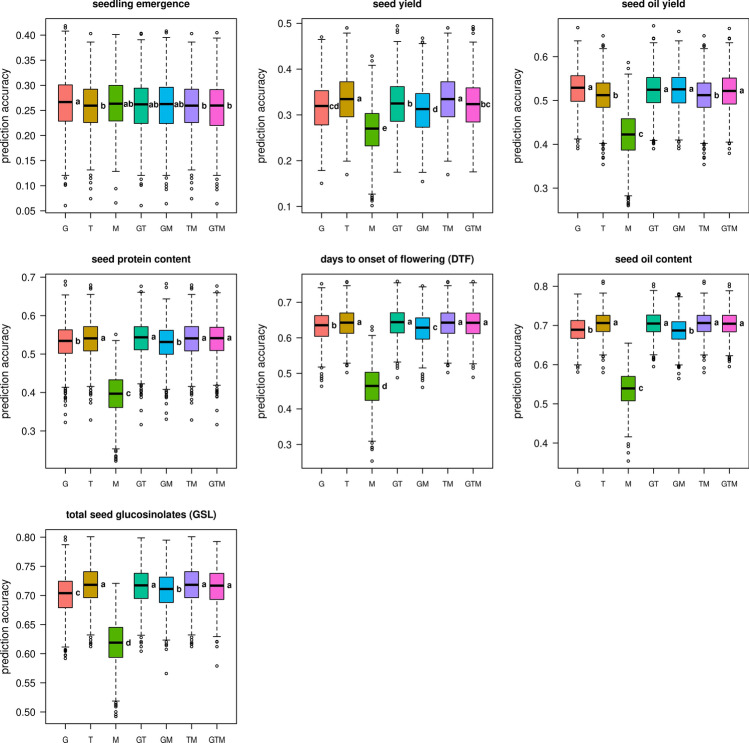
Prediction accuracies for reproducing kernel Hilbert space (RKHS) models. Predictions based on reproducing kernel Hilbert space regression (RKHS) models using Gaussian kernels. Predictors are denoted as: G = SNP-based genotype data, T = transcriptomic data, and M = metabolite data and their respective combinations GT, GM, MT and GTM. Letters beside the boxes indicate significant differences between predictor sets determined by a one-way ANOVA followed by a post-hoc Tukey’s multiple comparison test

**Fig. 5:**
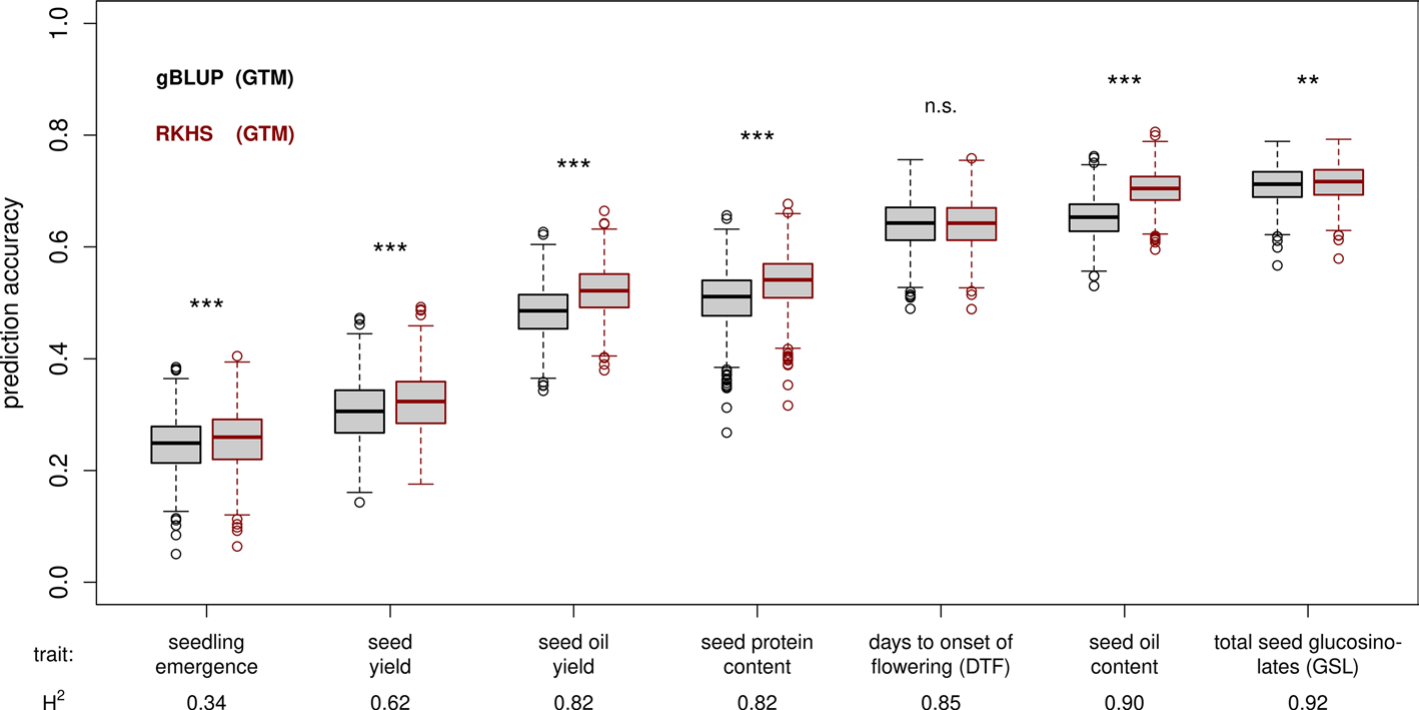
Comparison of reproducing kernel Hilbert space (RKHS) and gBLUP models. A cross-validation scheme with 1000 cycles was applied, separating the data set in a training set (75%) and a validation set (25%). Exemplarily, only the combination of all three omics data sets as predictors (genomic, transcriptomic and metabolite data; GTM) is shown. Asterisks above the plots indicate significant differences between gBLUP using additive relationship matrices and RKHS models determined by Welch’s two sample *t* test (alpha: * = 0.05; ** = 0.01 and *** = 0.001). The broad-sense heritability (H2) for each of the seven analysed traits is given at the bottom of the figure and the seven agronomic traits were sorted accordingly

In addition to gBLUP prediction (Habier et al. 2007, 2013; Goddard 2009), which has been used routinely as a base-line model in numerous studies, we employed a second model class, reproducing kernel Hilbert space regression based on Gaussian kernels (RKHS; Gianola and van Kaam 2008) for hybrid performance prediction (Fig. 4). RKHS exploits both the additive and to some extent non-additive effects among predictors. In general, prediction accuracies for the RKHS models followed a similar pattern as for the gBLUP models and clearly correlated with trait heritability. Similarly, the metabolite data individually yielded the lowest prediction accuracies. Interestingly, prediction accuracies of RKHS models including transcriptome data (T, GT, TM, GTM) were significantly higher than models not including transcripts (G, M, GM; Fig. 4) for the four traits, seed protein content, days to onset of flowering, seed oil content, and total seed GSL. In direct comparison, RKHS models stacking all three omics data layers were able to outperform gBLUP using additive relationship matrices for six out of the seven agronomic traits analysed: seedling emergence, seed yield, seed oil yield, seed protein content, seed oil content and total seed GSL content (Fig. 5, Data S2). Only for the trait days to onset of flowering no significant improvement was observed. Substantial increases in the mean prediction accuracy of up to 3.6% for seed oil yield, 3.0%, for seed protein content, and 5.3% for seed oil content were achieved using RKHS models. Even after combining the additive with the dominance and/or the epistatic genomic relationship matrices, RKHS models were in no case inferior to the corresponding gBLUP models (Figure S2, Data S2).

### Canola hybrids display substantial growth/biomass heterosis in the glasshouse

Five phenotyping experiments were performed in a climatized glasshouse with the 477 parental genotypes and a selection of 120 of the 950 hybrids (Knoch et al. 2020). These hybrids were selected with respect to their seed yield in the field trials whereby the 60 lines with highest overall seed yield and the 60 lines with lowest seed yield were selected. Hybrid biomass (FW) in the glasshouse showed a moderate positive correlation (*r* = 0.52; *p*-value = 7.76e−10) with hybrid seed yield in the field. The collection of phenotypic data for these hybrids in combination with the data of the 477 parental lines provided the basis to calculate best-parent heterosis (BPH) and mid-parent heterosis (MPH) values. Five traits with high heritability values were chosen: end-point biomass (fresh weight and dry weight), projected leaf area, estimated biovolume, and early plant height. Overall, far more positive than negative mid-parent values heterosis were detected, and even for BPH a trend towards positive heterosis was observed (Figure S3). For projected leaf area and estimated biovolume, calculations have been performed individually for all 21 days on the basis of BLUEs across all five phenotyping experiments. Strong positive, as well as negative MPH could be detected ranging from −29.0 to 64.6% for projected leaf area (Figure S3 a, b), from −40.8 to 122.5% for estimated biovolume (Figure S3 e, f) and from −22.9 to 43.3% for early plant height (Figure S3 i, j). Determined BPH values ranged from −36.4 to 47.9% for projected leaf area (Figure S3c, d), from −53.3 to 63.3% for estimated biovolume (Figure S3 g, h) and from −27.4 to 37.3% for early plant height (Figure S3 k, l). MPH values ranged from −27.2 to 72.3% for FW (Figure S3 m, n) and from −23.2 to 73.0% for DW, respectively (Figure S3 q, r). BPH values ranged from −39.6 to 41.3% for FW (Figure S3 o, p) and from −32.7 to 45.1% for DW (Figure S3 s, t), respectively. Distinct differences in the value distributions were detected when the 120 lines were grouped into ‘good’ and ‘bad’ hybrids with respect to seed yield data from the field trials. The set of ‘good’ hybrids displayed significantly higher MPH (Figure S3 b, f, j, n and r), as well as BPH values (Figure S3 d, h, l, p and t) for all five traits (Welch Two Sample *t* test, two sided, *p*-value = 1.31e−24) compared to the set of ‘bad’ hybrids. Hybrids originating from crosses with MS1 displayed significantly higher MPH values for leaf area and biovolume than crosses with MS2 (Data S1). In addition, performance differences regarding the agronomic traits were observed between the breeding pools when crossed to the two different MS lines (Data S3).

### Prediction of early growth-related traits and biomass in the glasshouse

Complementary to the field data, we also analysed and predicted biomass and biomass-related traits obtained from the glasshouse experiments in both parental lines and the selected hybrids using gBLUP and RKHS (Data S2). Both methods showed the same trends regarding the ranking of the predictor sets and combinations. In the following, we focus on gBLUP and early biomass production.

**Fig. 6:**
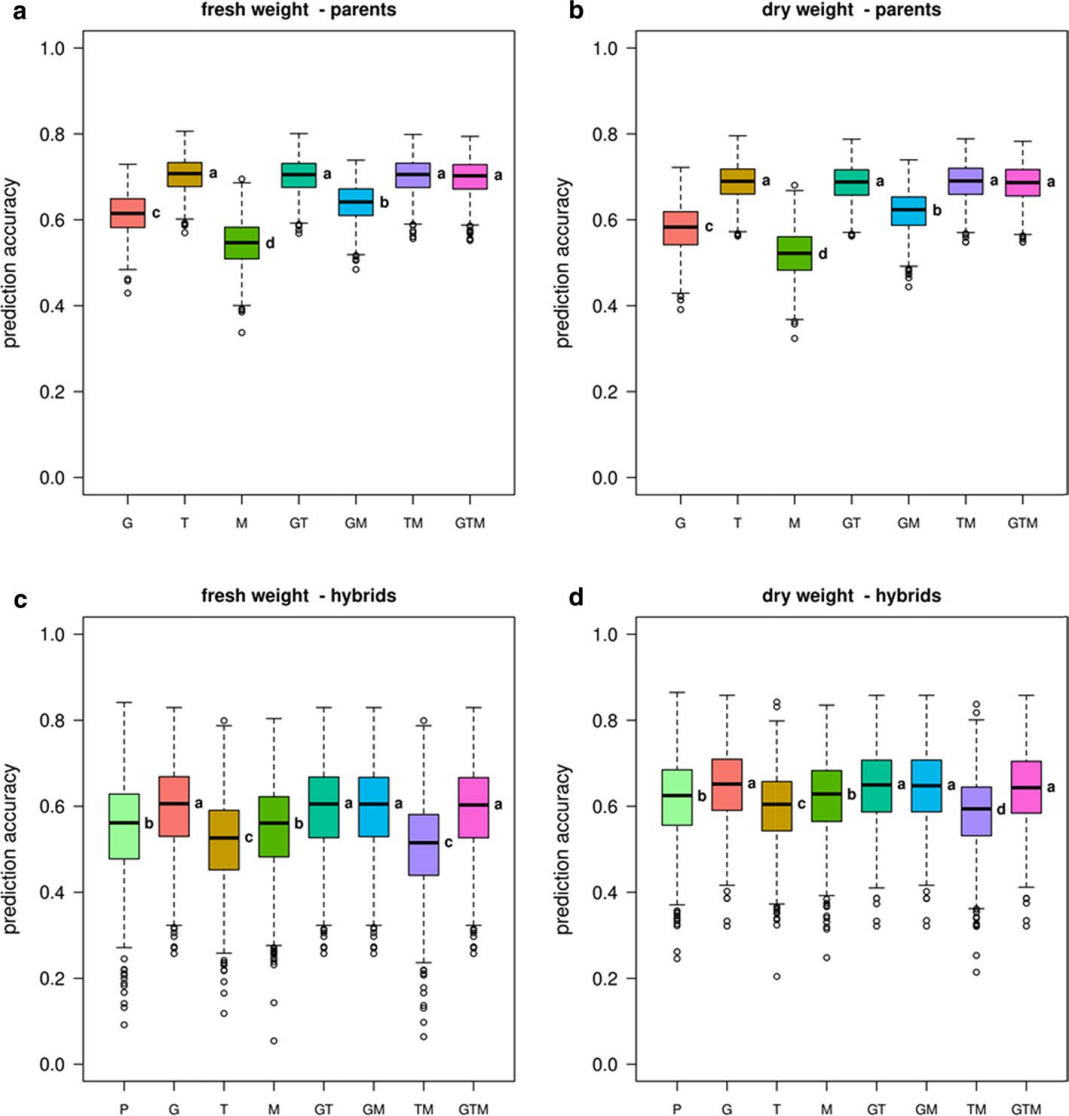
Prediction accuracies for early parent and hybrid biomass formed in the glasshouse. The summary of (genomic) best linear unbiased predictions (gBLUP) using additive relationship matrices for early plant biomass of (**a**, **b**) 477 parental lines and (**c**, **d**) the 120 hybrids is given as boxplots: fresh weight (left) and dry weight (right). The biomass data was obtained from the first to fourth and the fifth glasshouse phenotyping experiments for the parental lines and hybrids, respectively, at 28 DAS. Parental predictor data sets as: P = phenotype data, G = SNP-based genotype data, T = transcriptomic data, and M = metabolite data and their respective combinations GT, GM, MT and GTM. Letters beside the boxes indicate significant differences between predictor sets determined by a one-way ANOVA followed by a post-hoc Tukey’s multiple comparison test

Predicting parental biomass with parental data, no increased prediction accuracy was achieved by stacking all three omics data sets. However, a clear pattern with the highest prediction accuracies for the models including the transcriptome data could be observed for parental fresh and dry weight (Fig. 6a, b). The mean prediction accuracies obtained using the transcripts (FW = 0.705; DW = 0.687) were substantially higher than the accuracies reached by the genetic markers (FW = 0.613; DW = 0.579). Again, the metabolites represented the predictor data set with the lowest predictive abilities. Nevertheless, a combination of genomic and metabolomic data could significantly increase the prediction accuracy compared to pure genomic (SNP) markers.

Predicting hybrid biomass with parental data, it was hypothesised that the parental transcript and metabolite profiles might reflect more closely the hybrid biomass production, as parental lines and selected hybrids were grown in the same facilities under the same controlled environmental regime. Prediction analyses were performed with the same models and the predictor sets and their combinations as described above. In addition, parental phenotypic data (2436 image-derived phenotypic traits obtained over 21 days; Data S1) were also used as predictors. In contrast to the parental line performance, highest prediction accuracies (gBLUP) of 0.595 (FW) and 0.646 (DW) were achieved, using the genomic data set (Fig. 6c, d). The differences between the predictor sets were less pronounced than for the parental line performance, but the variation between the cross-validation cycles was substantially larger (Fig. 6). Notably, predicting hybrid biomass using the phenotypic data set of the parental lines yielded prediction accuracies comparable to those of the endophenotypes, and especially the parental metabolite profiles could predict early hybrid biomass with a higher efficiency than the transcriptome data (Fig. 6c, d).

In addition, hybrid projected leaf area, estimated biovolume, and early plant height between 6 and 27 DAS (Knoch et al. 2020) were used as response variables (Figure S4) to evaluate the stability of predictions for time series data. Predictions for biomass-related traits with gBLUP models and all predictor sets combined yield low prediction accuracies at early time points, but increase over time and reach saturation at a value of approximately 0.6 (Figure S4; Data S2). We also predicted heterosis for biomass and growth-related traits. However, MPH and BPH values could only be predicted with low to moderate accuracy (Data S2). The highest mean accuracy could be determined for projected leaf area BPH (0.577) at 10 DAS, and a tendency towards higher prediction accuracies for earlier time points could be observed.

## Discussion

We evaluated the benefits of combining large parental omics datasets for the prediction of hybrid performance using different statistical models in spring-type oilseed rape from an elite breeding programme, thus providing important breeding strategy information. For the generation of omics data sets, the time point at 14 days after sowing (DAS) was chosen for sampling as data obtained at an early growth stage harbour important information for the prediction of hybrid performance (Riedelsheimer et al. 2012). Previous studies extensively elaborated on the effectiveness of different prediction methods (Xu et al. 2016; Momen et al. 2018; Li et al. 2020a), often showing that the impact of the different prediction models was negligible and that prediction abilities strongly depended on the genetic architecture of the traits. Hence, only two types of prediction models were compared: classical genomic best linear unbiased predictions (gBLUP) as current standard, and reproducing kernel Hilbert space regression (RKHS) based on Gaussian kernels with the potential advantage to capture also non-additive effects among predictors. The parental omics sets comprised array-derived genotype data, including single nucleotide polymorphisms (SNPs) and copy number variations (CNVs), global transcriptome (RNA-Seq) profiles, primary metabolite (GC–MS) profiles, as well as detailed high-throughput image-derived phenotype data. CNVs and presence-absence variations (PAVs) have been shown to carry complementary information to SNP markers, and to yield additional associations with phenotypic traits (Gabur et al. 2020). A large number of deletions but only a few duplications were detected in comparison to the reference genome, which is consistent with previous studies in canola (Cao and Schmidt 2013; Zou et al. 2018). The combination of SNP and CNV markers in our gBLUP models did significantly increase prediction accuracies for three agronomic traits compared to pure SNP markers. Seed yield showed a considerable gain in prediction accuracy for both gBLUP and RHKS models. The beneficial effect of adding CNVs was negligible in the stacked models, indicating that the relevant information carried by the CNVs might also be covered by the transcript data.

Prediction accuracies strongly correlated with trait heritability, which is consistent with previous observations that prediction accuracies depend on many factors including trait heritability, genetic complexity of the trait, density of genetic markers, and quality of the input phenotype data (Liu et al. 2018b; Zhang et al. 2019). Phenotypic traits of low or intermediate heritability like seedling emergence (*H*^2^ = 0.34) or seed yield (*H*^2^ = 0.62), could only be predicted with low to moderate prediction accuracy. In contrast, traits with a high heritability like seed oil content (*H*^2^ = 0.90) and total seed GSL content (*H*^2^ = 0.92) were predicted with high accuracy. The median prediction accuracies, ranging from 0.25 (seedling emergence) to 0.72 (total seed GSL content), reflected the differences in genetic architecture of the traits. Seed yield is a highly polygenic trait heavily influenced by $GxE$ interactions (Marjanović-Jeromela et al. 2011; Escobar et al. 2011). Total seed GSL content, although noticeably influenced by environmental factors (He et al. 2018), is controlled by a relatively small core set of biosynthesis and degradation genes and regulators (Grubb and Abel 2006; Halkier and Gershenzon 2006; Ishida et al. 2014). As shown previously (Liu et al. 2018b), the number of predictors also affected prediction accuracy. In our study, the metabolite data contained the smallest number of predictors and yielded the lowest prediction accuracies. Although key substances of the primary metabolism such as intermediates of the TCA cycle, amino acids, organic acids, sugars and sugar alcohols were included, the polar metabolites quantified represent only a fraction of the whole metabolome. Xu et al. (2016) also reported poor prediction accuracies when using only a subset of 100 metabolites compared to the full set of 1000 metabolites.

The genetic (13,201 SNPs) and the transcriptome (19,479 transcripts) data showed particularly high predictive abilities. These results indicate that the number of genetic/transcriptomic markers was sufficient to cover most causative genes and their effects. This observation is supported by a recent study in maize (Westhues et al. 2017), where SNP-based predictive abilities reached a plateau after using 5000 equally spaced markers. However, reducing the number of genetic markers and transcripts to the number of metabolites (*n* = 154) did not influence the ranking of the predictors in our study; the polar leaf metabolites still show the lowest prediction accuracies. It seems likely that the analysed set of metabolites and/or their low number does not sufficiently cover the pathways associated with the traits of interest and underlying genetic factors and their interactions, or that a higher variability in the metabolite levels compared to the other omics data sets causes the overall lower prediction accuracies. These observations are in accordance with results of Westhues et al. (2017), who also observed trait-dependent prediction accuracies, and overall lower prediction accuracies for leaf metabolites compared to genetic markers. Similarly, Riedelsheimer et al. (2012) reported that prediction accuracies for metabolites were on average 6.7% lower compared to SNP data. Zhao et al. (2015) also reported in a study in wheat that integration of metabolomic data did not result in superior predictions for grain yield compared to genomic prediction. However, they integrated only a very small number of 35 metabolites in their predictive models. In contrast, a recent study analysing seed yield, 1000 grain weight, number of grains per panicle, and number of tillers per plant in hybrid rice, identified the combination of genetic and metabolic markers together with BLUP models as most promising strategy for prediction of hybrid performance (Wang et al. 2019). Moreover, Dan et al. (2020) were successful in predicting yield heterosis in rice using 3746 metabolic analytes detected by liquid chromatography–mass spectrometry (LC–MS).

Although the genotypic and the transcriptome data sets were comparable in their number of features, the transcriptome data alone yielded moderately but significantly increased prediction accuracies for five out of seven agronomic traits analysed. The maximum gain was observed for total seed GSL content with 2.6%. Still, the increase of even a few percent in prediction accuracy can have a substantial impact for plant breeding. A relevant difference between genetic markers and transcripts is that SNPs are binary, while transcript data are quantitative with the potential of large dynamic ranges. Thus, the information content per transcript is much deeper than per SNP marker. The observed increase suggests that the transcriptome data covers biological information derived from different regulatory levels not captured by the genetic markers. This is in line with Westhues et al. (2017) who also obtained better predictions using transcriptomic data and showed in an eQTL analysis that transcriptomic data integrate cis and trans effects of the expressed genes. The superior performance of transcriptome data is further confirmed by our cross-validation approach using 10,000 features for both sets. The transcripts significantly outperformed the genetic markers for five (DTF, total seed GSL content, seed protein content, seed oil content and seed yield) of the seven traits analysed. However, further stacking of omics data sets did not significantly increase prediction accuracies. Hence, the initial hypothesis that stacking of diverse omics profiles can improve hybrid predictions could only be partially supported. A study by Xu et al. (2017) demonstrated a better performance of genomic prediction than transcriptomic or metabolomic predictions in maize. In contrast, Westhues et al. (2017) obtained a significant improvement of predictive abilities by integration of endophenotypes with genetic markers. Overall, these findings suggest strong differences in the valorisation potential of omics data between species, traits and populations.

We also predicted hybrid and per se performance parameters expressed in the glasshouse. A selection of 120 hybrids was grown and phenotyped in a separate experiment under the same conditions as the 477 parental lines, which allowed us to compare early vegetative biomass and growth-related traits in both groups. Hybrid FW was overall moderately correlated with the FW of the parental pollinators (*r* = 0.48), also reflected in the ability of the parental phenotype data to predict hybrid biomass in the glasshouse. This correlation differed substantially between hybrids with ‘good’ (*r* = 0.62) and ‘bad’ (*r* = 0.21) seed yield in the field, pointing to a link between the per se performance of parental lines and biomass for at least a subset of the hybrids. These findings and the positive correlation between hybrid seed yield and hybrid biomass (*r* = 0.52) indicate that biomass production is positively linked to seed yield in canola, which is in concordance with previous studies (Basunanda et al. 2010; Zhao et al. 2016). Li et al. (2020b) described a colocalization of QTL for growth-related traits and QTL for final yield. The future exploration of the link between early vegetative biomass production and seed yield might represent a novel strategy for genetic improvement of rapeseed, in particular for the spring type. Moreover, a significant effect of the male-sterile mother lines on biomass production was observed. Hybrids with MS1 as female parent, which displayed significantly higher biomass than MS2, produced overall larger plants in comparison to hybrids originating from crosses with MS2. The two male-sterile testers were selected as representatives of two divergent subgroups of breeding pool 1. However, in oilseed rape heterotic groups (Melchinger and Gumber 1998) are not yet well established, and genetic distances between pools are not as large (Qian et al. 2007; Rincent et al. 2014) as for instance in the flint and dent populations of European maize (Younas et al. 2012; Liu et al. 2019). This can be attributed in particular to a less intensive and shorter breeding history of canola compared to maize (Chalhoub et al. 2014; Hu et al. 2019).

Parental metabolite profiles predicted hybrid performance in the glasshouse substantially better than hybrid performance in the field, as indicated by smaller differences in prediction accuracy between the predictor data sets. This could be attributed to the fact that parental lines and hybrids were grown in the same environment and were in a comparable physiological state, i.e. early vegetative development (14 DAS for metabolite profiling and 28 DAS for phenotype data). Prediction accuracies for ‘projected leaf area’ and ‘estimated biovolume’, scored between 6 and 21 DAS, were relatively low for early time points, but increased over time and reached saturation at a value of approximately 0.6 for both traits. This observation may reflect maternal effects in the earlier phases of plant growth and/or environmental effects during seed formation. The effects diminish as soon as plants establish new leaves and shift from drawing nutrients from the storage tissue to own photosynthesis, as observed in Arabidopsis seeds and young seedlings (Meyer et al. 2012).

Hybrid performance is known to be driven by a mix of additive, dominance, and epistatic effects, as shown in an immortalized F_2_ rapeseed population by Liu et al. (2017). Combining either a dominance or an epistatic genomic relationship matrix with our additive gBLUP models lead to a significant gain in prediction accuracy for several traits, which is in contrast to investigations in maize (Li et al. 2020a) and rapeseed (Werner et al. 2017). The prediction accuracies of four traits could be further improved by adding the additive relationship matrices calculated from transcript and metabolite data to the model. Since no established procedures exist for coding epistasis or dominance using quantitative data, we employed reproducing kernel Hilbert space regression (RKHS; Gianola and van Kaam 2008), which is able to exploit non-additive effects among markers. In direct comparison to additive gBLUP models, the usage of RKHS could substantially improve the prediction accuracies for most agronomic traits, up to 5.3% in case of seed coil content using the combination of genomic and transcriptomic data. The higher prediction accuracies in the RKHS models and in gBLUP models including epistatic and/or dominance matrices, indicate that at least for some of the agronomic traits epistatic interactions contribute to trait manifestation. It has been shown that epistasis plays a major role in rapeseed yield formation (Luo et al. 2017), and, together with heterozygous loci, influences yield heterosis (Radoev et al. 2008). Epistatic interactions of loci, especially additive x additive epistasis, accounting for a high proportion of variance were also described for a number of yield-related traits in canola, including biomass yield, days to onset of flowering, plant height, branch number, harvest index, seed oil content and seed protein content (Zhao et al. 2006; Shi et al. 2011; Li et al. 2012; Würschum et al. 2013). Dominant effects were found to account for only a small proportion of variance, and QTL and epistatic interactions clustered on several chromosomes (Shi et al. 2011). However, both individual QTL and epistatic interactions explained on average less than 10% of phenotypic variance. As only two epistatic interactions of seed yield were detected in different environments, the authors suggested that epistatic interactions of yield-related traits are extremely sensitive to environmental variation. Xu et al. (2014) evaluated prediction models for hybrid rice including main effect models, and models incorporating dominance and epistasis. In their study, there was no noticeable improvement of the epistatic model over the additive model using real-life data. However, they could demonstrate in simulation studies that predictions can be further improved by incorporating dominance and epistasis into the model. Another recent study of diverse populations in hybrid maize suggests that considering dominance effects and gene annotations can improve genomic predictions, in particular for plant height (Ramstein et al. 2020).

In our study, RKHS was in no tested case inferior to gBLUP. However, the differences between models were in most cases only subtle compared to differences between different traits. Trait heritability, genetic complexity of the traits, and quality and size of input phenotype data seem more important than the prediction model, as previously reported by Werner et al. (2017). Nevertheless, RKHS or other models incorporating non-additive/epistatic effects like EGBLUP (Jiang and Reif 2015) or Bayesian models (Habier et al. 2011; Yang and Tempelman 2012; Werner et al. 2017; Fikere et al. 2018) are preferable to gBLUP only using an additive relationship matrix for prediction of hybrid performance, especially if the genetic architecture of the trait is unknown.

In current breeding programmes, pedigree and genomic data are typically utilised for routine analyses and represent a well-established alternative to large numbers of test crosses. To further increase the accuracy of predictions, it is of major interest to test and compare the predictive abilities of omics data for different traits and whether a combination of predictors provides higher predictive ability. In the present study, transcriptomic predictions achieved higher prediction accuracies for five out of seven agronomic traits compared to genetic markers. However, for both metabolomic and transcriptomic data, the expenses and workload involved in their generation often outweigh their potential gain. Thus, genomic data, which have the best cost–benefit ratio, can currently be considered sufficient for most breeding programmes and traits. Supplementing SNP by CNV data, called from the same array or sequencing data, can provide an opportunity to increase prediction accuracy, as shown for seed yield in our study. Inclusion of endophenotypes in predictive models may become attractive in plant breeding, if costs can be substantially reduced by streamlining sampling and processing approaches and by missing data imputation (Westhues et al. 2019). They also represent an interesting substitute for traits that are difficult and/or expensive to score in the field. The inclusion of environmental data and their interaction with omics data can potentially improve trait predictabilities, as shown by a recent study in pearl millet (Jarquin et al. 2020). A study on hybrid prediction in grain maize illustrated that including historic phenotypic data for training improves genomic prediction and enables optimization of hybrid variety development (Schrag et al. 2019). Two other studies, Westhues et al. (2017) and de Abreu e Lima et al. (2017), described promising results regarding the utilisation of metabolites, in particular from young roots. In addition to the primary metabolites quantified by GC–MS, the utilisation of energy metabolites and/or lipids by targeted and untargeted metabolic profiling using LC–MS might represent a valuable future strategy to broaden the number and type of predictive features to increase prediction accuracies. Furthermore, time series data and data collected from different tissues and organs should be screened for their potential to improve the prediction of hybrid performance. As prediction accuracy is affected by the density of genetic markers, a promising strategy might be to call SNPs de novo from the transcriptome data to increase the number of predictors and coverage of genetic factors associated with the trait of interest in appropriate populations. A recent study in semi-winter rapeseed demonstrated that already low-density marker sets comprising a few hundred to thousand markers enable high prediction accuracies in breeding populations with strong LD (Werner et al. 2018). As reviewed by Washburn et al. (2020), key improvements of genomic prediction might come from high‐throughput phenotyping, the use of molecular phenotypes and/or component traits, machine learning methodologies, and replacing individual genetic markers with high‐quality haplotype data.

We conclude that using downstream omics data, in particular transcript abundancies, carry important information beyond genomic data, which can be exploited to improve prediction accuracy. However, genetic markers are in general sufficient for prediction of agronomic traits and represent, at least at the moment, the most cost-efficient predictor sets. Importantly, our study reveals the advantage of reproducing kernel Hilbert space regression based on Gaussian kernels for hybrid prediction in canola breeding.

## Supplementary Information

Below is the link to the electronic supplementary material.

Supplementary material 1 (DOCX 1480 kb)

Supplementary material 2 (XLSX 156990 kb)

Supplementary material 3 (XLSX 8863 kb)

Supplementary material 4 (PDF 20 kb)

## Data Availability

Data will be made available upon request by the authors, if not stated otherwise.
