# Supplementary material for: Multi-omics-based prediction of hybrid performance in canola
Source: Theor Appl Genet. 2021 Feb 1;134(4):1147–65. doi: 10.1007/s00122-020-03759-x (PMC7973648; doi:10.1007/s00122-020-03759-x)
Supplement: Supplementary file 1 — Supplementary material 1 (DOCX 1480 kb) [file 122_2020_3759_MOESM1_ESM.docx]

**Multi-omics-based prediction of hybrid performance in canola**

Dominic Knoch^1*^, Christian R. Werner^2^, Rhonda C. Meyer^1^, David Riewe^3,1^,

Amine Abbadi^4,5^, Sophie Lücke^5^, Rod J. Snowdon^6^ and Thomas Altmann^1^

^1^ Leibniz Institute of Plant Genetics and Crop Plant Research (IPK), Department of Molecular Genetics, 06466 Seeland OT Gatersleben, Germany

^2^ The Roslin Institute, University of Edinburgh, Easter Bush, Midlothian, EH25 9RG, Scotland, United Kingdom

^3^ Julius Kühn Institute (JKI) – Federal Research Centre for Cultivated Plants, Institute for Ecological Chemistry, Plant Analysis and Stored Product Protection, 14195 Berlin, Germany

^4^ NPZ Innovation GmbH, Hohenlieth, 24363 Holtsee, Germany

^5^ Norddeutsche Pflanzenzucht Hans-Georg Lembke KG, Hohenlieth, 24363 Holtsee, Germany

^6^ Department of Plant Breeding, IFZ Research Centre for Biosystems, Land Use and Nutrition, Justus Liebig University, Heinrich-Buff-Ring 26-32, 35392 Giessen, Germany

**^*^** To whom correspondence should be addressed: Dominic Knoch

E-Mail: [knochd@ipk-gatersleben.de](mailto:knochd@ipk-gatersleben.de) ORCID [0000-0002-9362-3105](http://orcid.org/0000-0002-9362-3105)

Phone: +49 039482 5 809

**SUPPORTING INFORMATION**

Figure S1. Properties of predictor data sets displayed by multivariate analyses

Figure S2. Stacking additive, dominance and epistatic genomic relationship matrices

Figure S3. Hybrids display strong heterosis for biomass and growth related traits

Figure S4. Prediction of growth-related traits in the hybrids

Data S1. Experimental design, hybrid phenotypes and -omics predictor sets.

Data S2. Comprehensive overview of prediction accuracies.

Data S3. Performance differences between pools when crossed to MS lines

# SUPPORTING INFORMATION


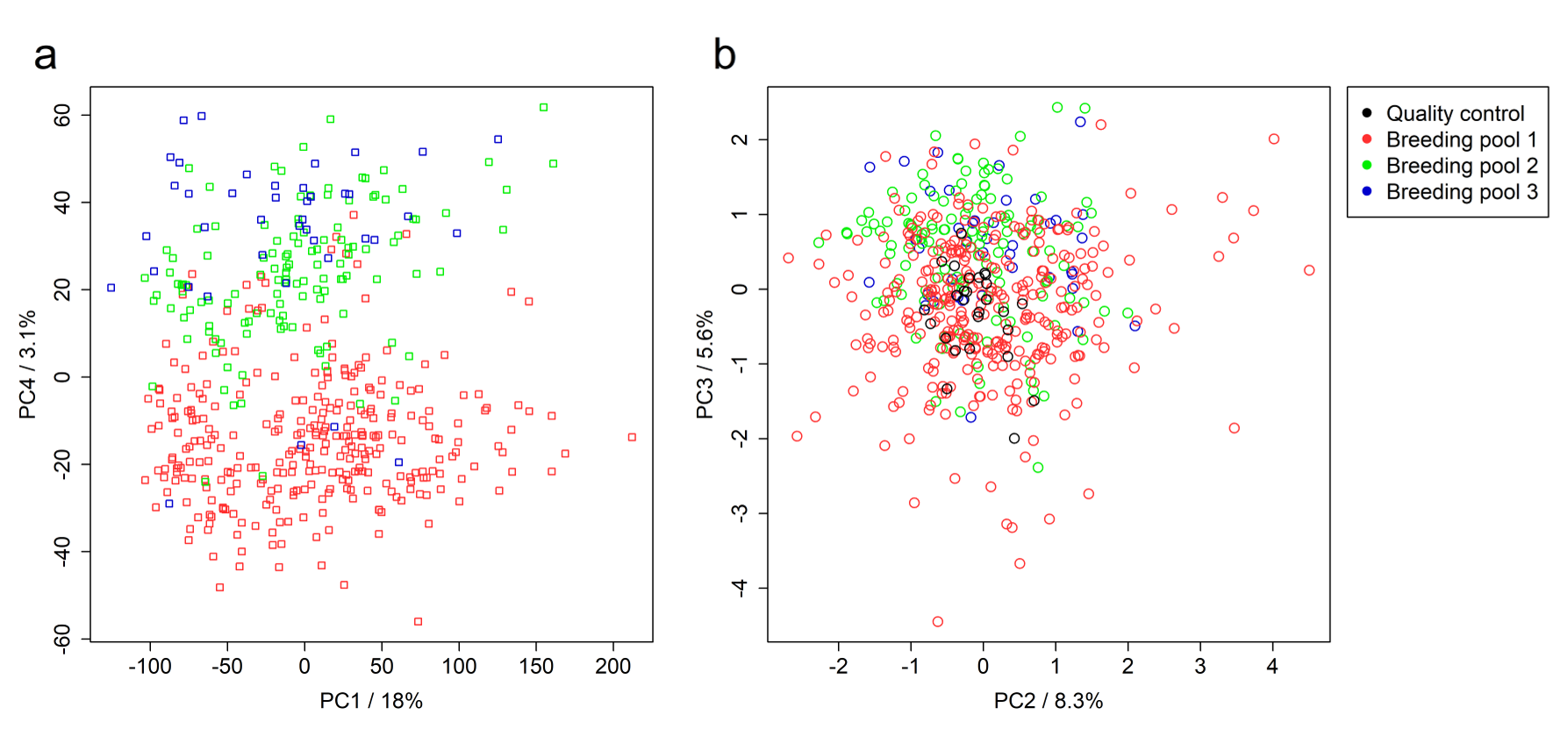


**Figure S1. Properties of predictor data sets displayed by multivariate analyses**

A principal component analysis on filtered transcript data (median tpm ≥ 5) for the 477 parental genotypes is shown in panel **a**. Transcript data were centred and scaled (z-scores). The PCA calculation was done by singular value decomposition (svd) of the data matrix. The first four PCs explained 18 %, 9.7 %, 3.8 % and 3.1 % of variance, respectively. A scatter plot of PC1 and PC4 with samples coloured according to their affiliation to the three breeding pools is shown.
A principal component analysis on normalised polar primary metabolite data analysed by
GC-MS analysis is displayed in panel **b**. Metabolites were centred and scaled and z-scores for each metabolite were generated (negative controls were excluded from the PCA).
PCA calculations were performed by an iterative method using a Bayesian model to handle missing values. The first four PC groups contribute 23 %, 8.4 %, 5.8 %, and 4.9 % to the metabolic variance, respectively. The scatter plot of PC2 and PC3 with the proportions of explained variance given on the axes. The colour key refers to the quality control pools (n= 27); the four reference lines (‘Achat’, ‘Campino’, ‘MS1’, ‘MS2’, each n= 7), and the 477 pollinators grouped by the three breeding pools (‘breeding pool 1, ‘breeding pool 2, and ‘breeding pool 3), respectively.


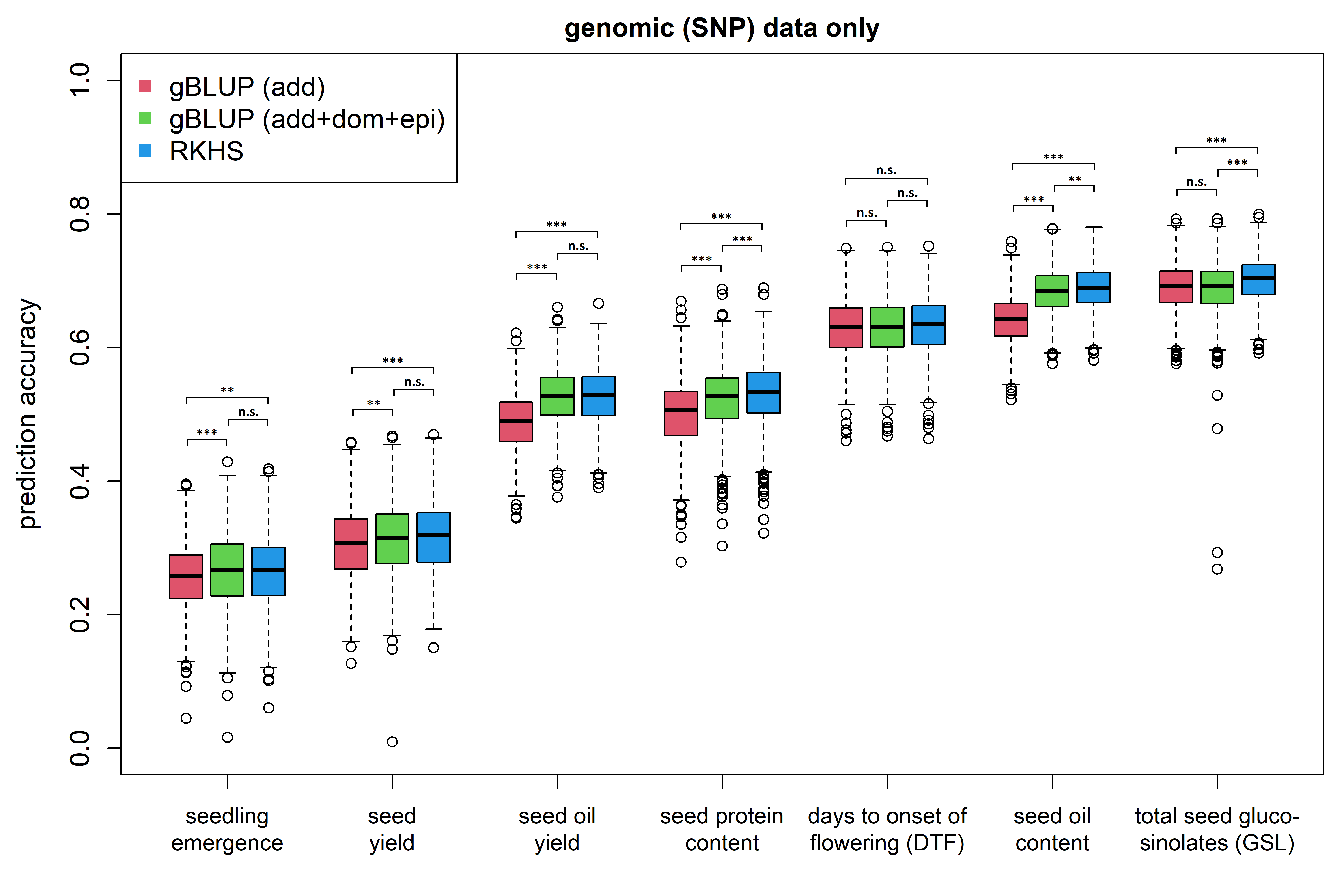


Figure S2. Stacking additive, dominance and epistatic genomic relationship matrices

Comparison between (genomic) best linear unbiased predictions (gBLUP) using an additive relationship matrix (red), a combination of additive, dominance and a second order additive x additive epistatic (green) genomic relationship matrices, and predictions based on reproducing kernel Hilbert space regression (RKHS) models using a Gaussian kernel (blue) for hybrid field performance. All matrices were based on genetic (SNP) marker data. The prediction accuracies of the models were defined as the correlation between the true and the predicted phenotypic values. A cross-validation scheme with 1,000 cycles was applied, separating the data set in a training set (75 %) and a validation set (25 %). Asterisks over the boxes indicate significant differences between the models determined by a one-way ANOVA followed by a post-hoc Tukey’s multiple comparison test (multiple comparisons adjusted *p*-value: *** < 0.001, ** < 0.01, * < 0.05, and n.s = not significant). A full comparison of the additive, dominance and epistasis relationship matrices and their combinations is given in Data S2.


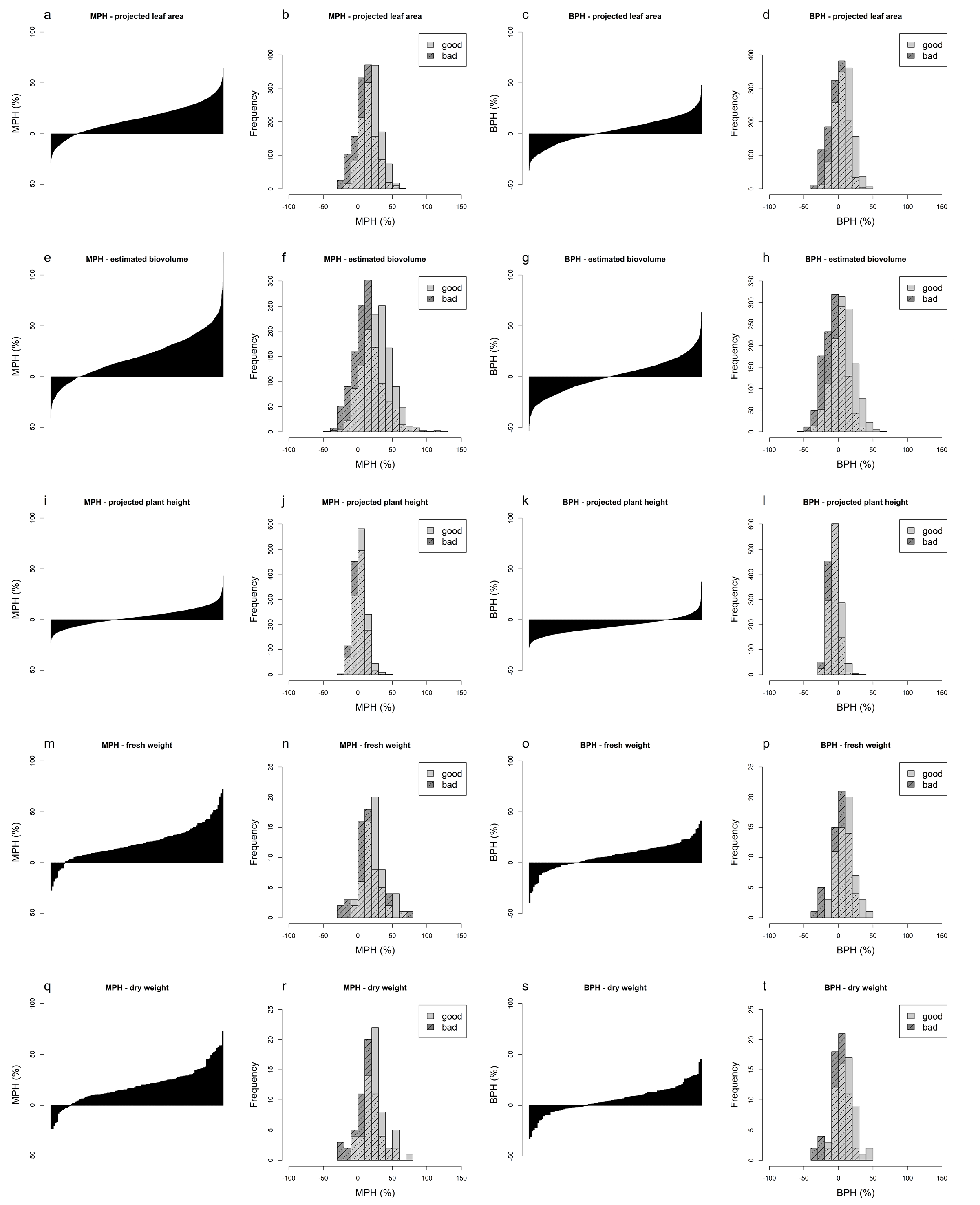


Figure S3. Hybrids display strong heterosis for biomass and growth related traits

Overview figure of mid-parent (MPH) and best-parent (BPH) heterosis for five selected traits assessed through phenotyping of glasshouse-cultivated plants: projected leaf area, estimated biovolume, early plant height, fresh weight (FW), and dry weight (DW). The panels **a**, **e**, **I, m** and **q** display bar plots of MPH and the panels **b**, **f**, **j, n** and **r** show MPH values as histograms with hybrids distinguished by ‘good’ and ‘bad’ seed yield in the field trials, indicated by partially transparent dark and light grey, respectively. The panels **c,** **g,** **k, o** and **s** display the BPH values calculated for the same hybrids and traits. The panels **d**, **h**, **l, p** and **t** show the histograms for BPH traits. FW and DW were determined at 28 DAS. The MPH and BPH values for projected leaf area, estimated biovolume, and early plant height are shown combined for all 21 days of phenotyping (6 to 27 DAS).


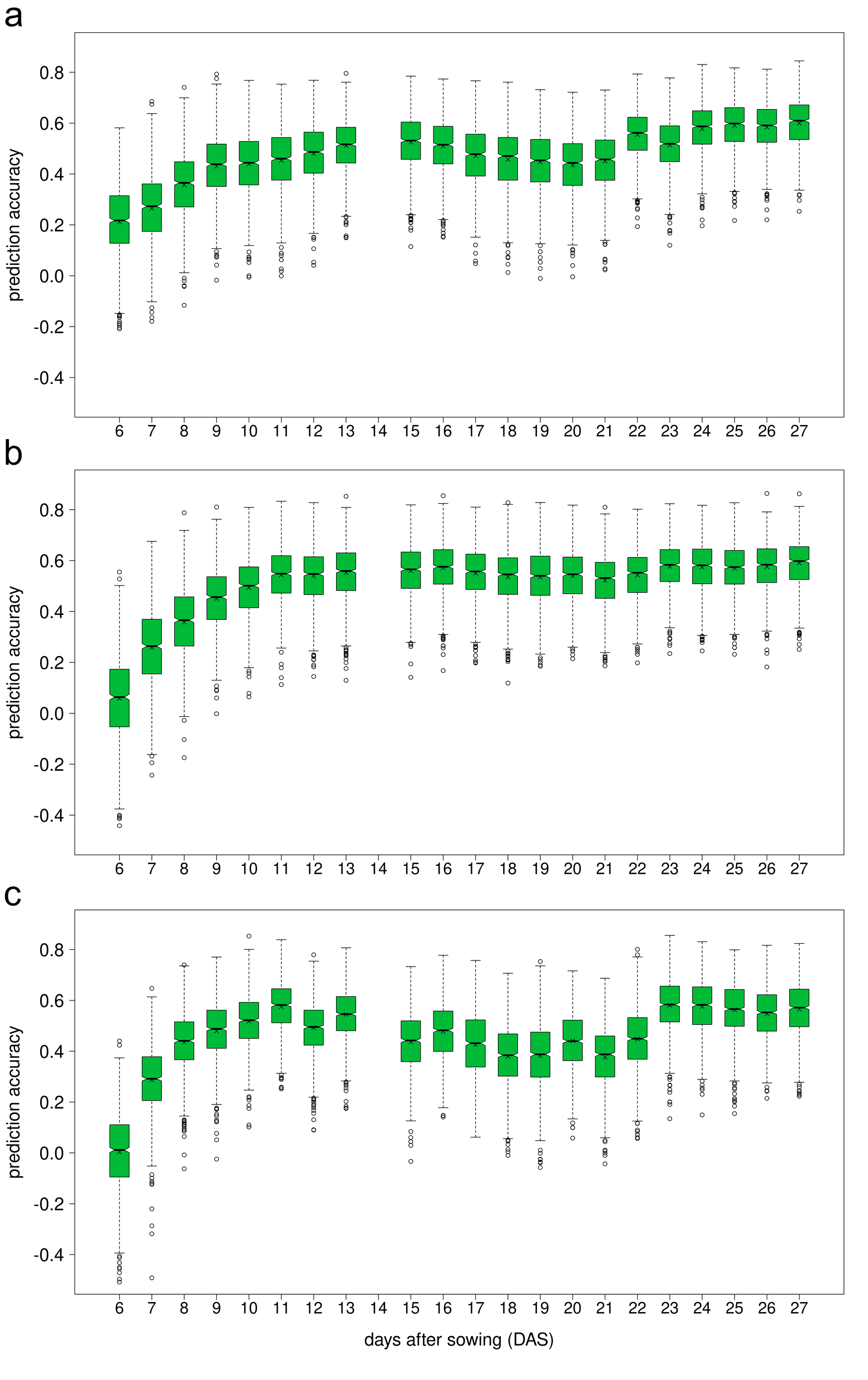


Figure S4. Prediction of growth-related traits in the hybrids

Shown are (genomic) best linear unbiased predictions (gBLUP) using additive relationship matrices of time series data for **a** projected leaf area, **b** estimated biovolume and **c** early plant height for the set of 120 hybrids as boxplots. The prediction accuracies of the models were defined as the correlation between the true and the predicted phenotypic values. A cross-validation scheme with 1,000 cycles was applied, separating the data set in a training set (75 %) and a validation set (25 %). The analysis was performed using the combination of all three -omics data sets as predictors. GTM: SNP-based genotype (G) + transcriptomic (T) + metabolite (M) data. The paternal -omics data sets (T & M) were obtained from the parental lines of the hybrids cultivated in the same glasshouse as the hybrids under the same environmental conditions, but in a different experiment.
