# Supplementary material for: Multi-omics-based prediction of hybrid performance in canola
Source: Theor Appl Genet. 2021 Feb 1;134(4):1147–65. doi: 10.1007/s00122-020-03759-x (PMC7973648; doi:10.1007/s00122-020-03759-x)
Supplement: Supplementary file 4 — Supplementary material 4 (PDF 20 kb) [file 122_2020_3759_MOESM4_ESM.pdf]

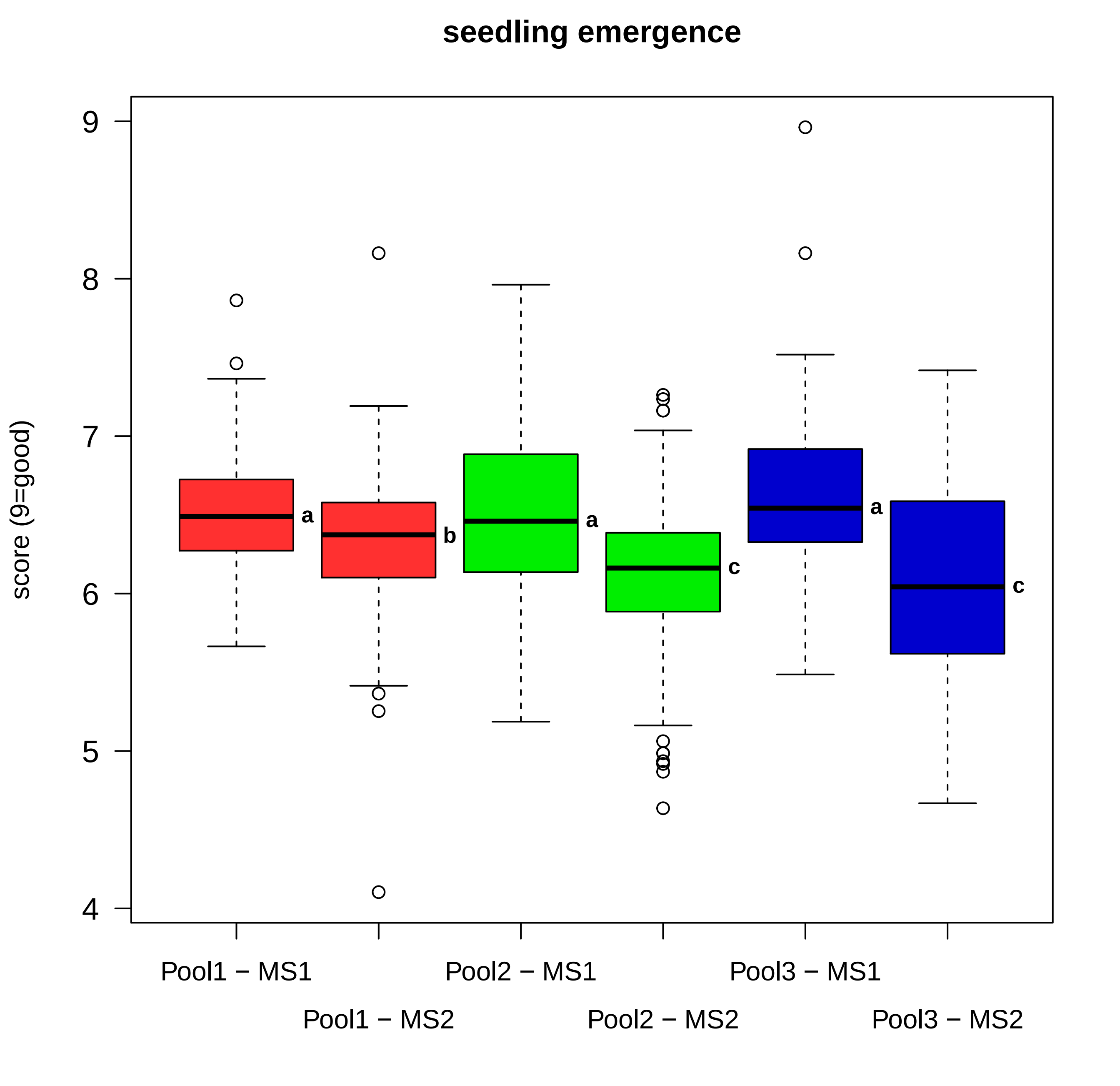
seedling emergence
score (9=good)
9
8
7
6
5
4
a
b
a
c
a
c
Pool1 − MS1
Pool1 − MS2
Pool2 − MS1
Pool2 − MS2
Pool3 − MS1
Pool3 − MS2

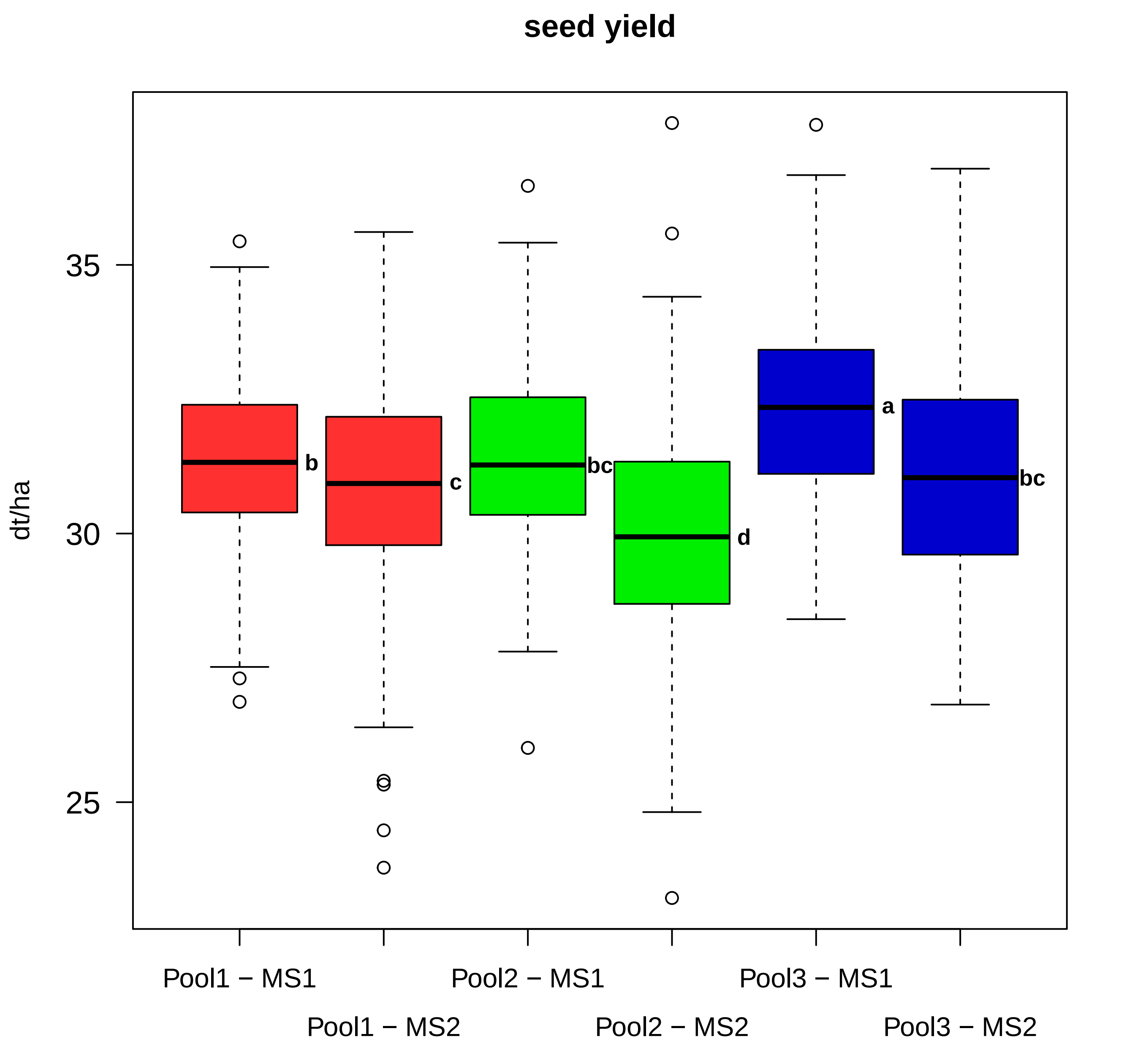

seed yield
dt/ha
35
30
25
b
c
bc
d
a
bc
Pool1 − MS1
Pool1 − MS2
Pool2 − MS1
Pool2 − MS2
Pool3 − MS1
Pool3 − MS2

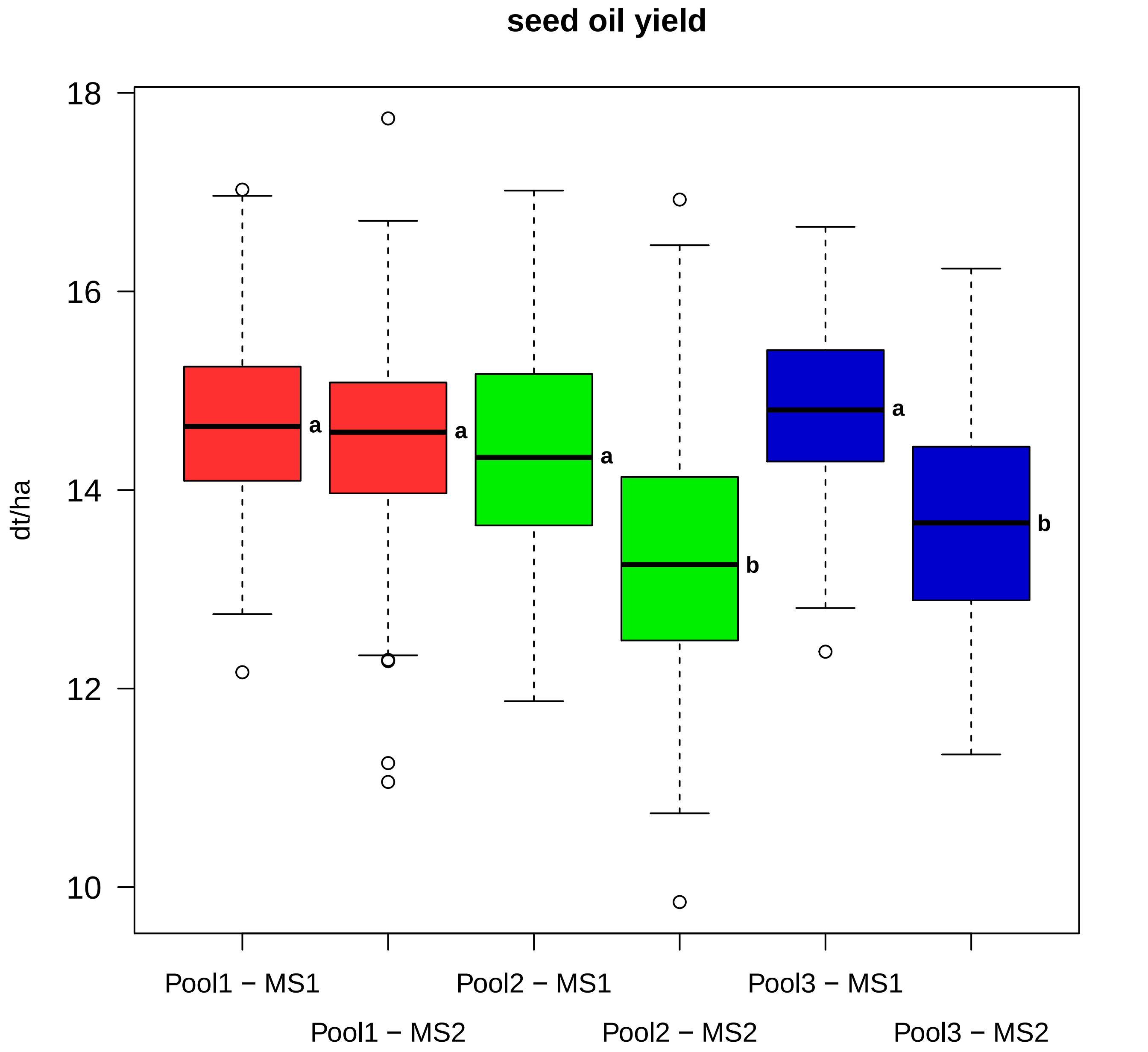
seed oil yield
dt/ha
18
16
14
12
10
a
a
a
b
a
b
Pool1 − MS1
Pool1 − MS2
Pool2 − MS1
Pool2 − MS2
Pool3 − MS1
Pool3 − MS2

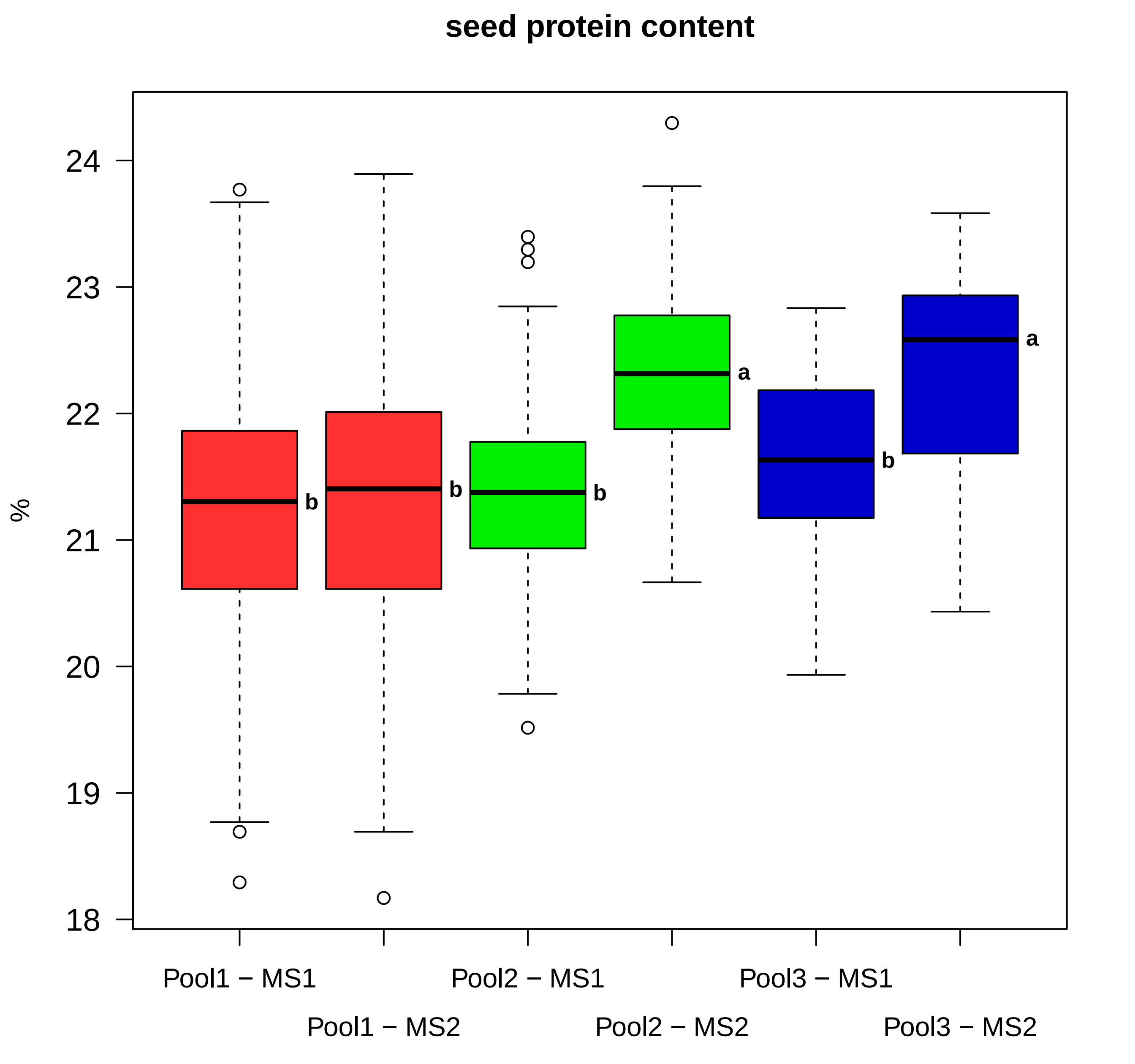
seed protein content
%
24
23
22
21
20
19
18
b
b
b
a
b
a
Pool1 − MS1
Pool1 − MS2
Pool2 − MS1
Pool2 − MS2
Pool3 − MS1
Pool3 − MS2

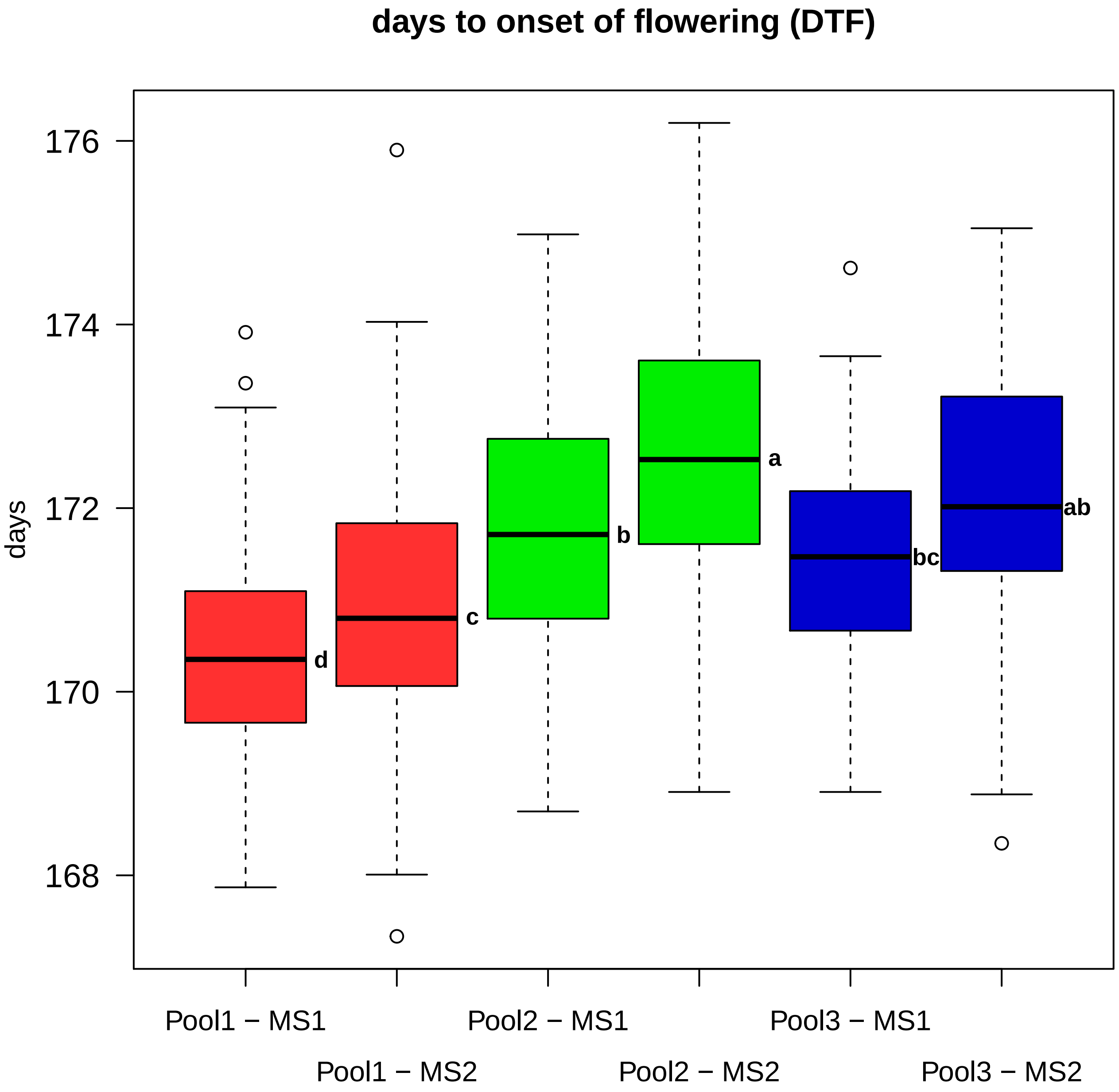

days to onset of flowering (DTF)
days
176
174
172
170
168
a
ab
b
bc
c
d
Pool1 − MS1
Pool1 − MS2
Pool2 − MS1
Pool2 − MS2
Pool3 − MS1
Pool3 − MS2

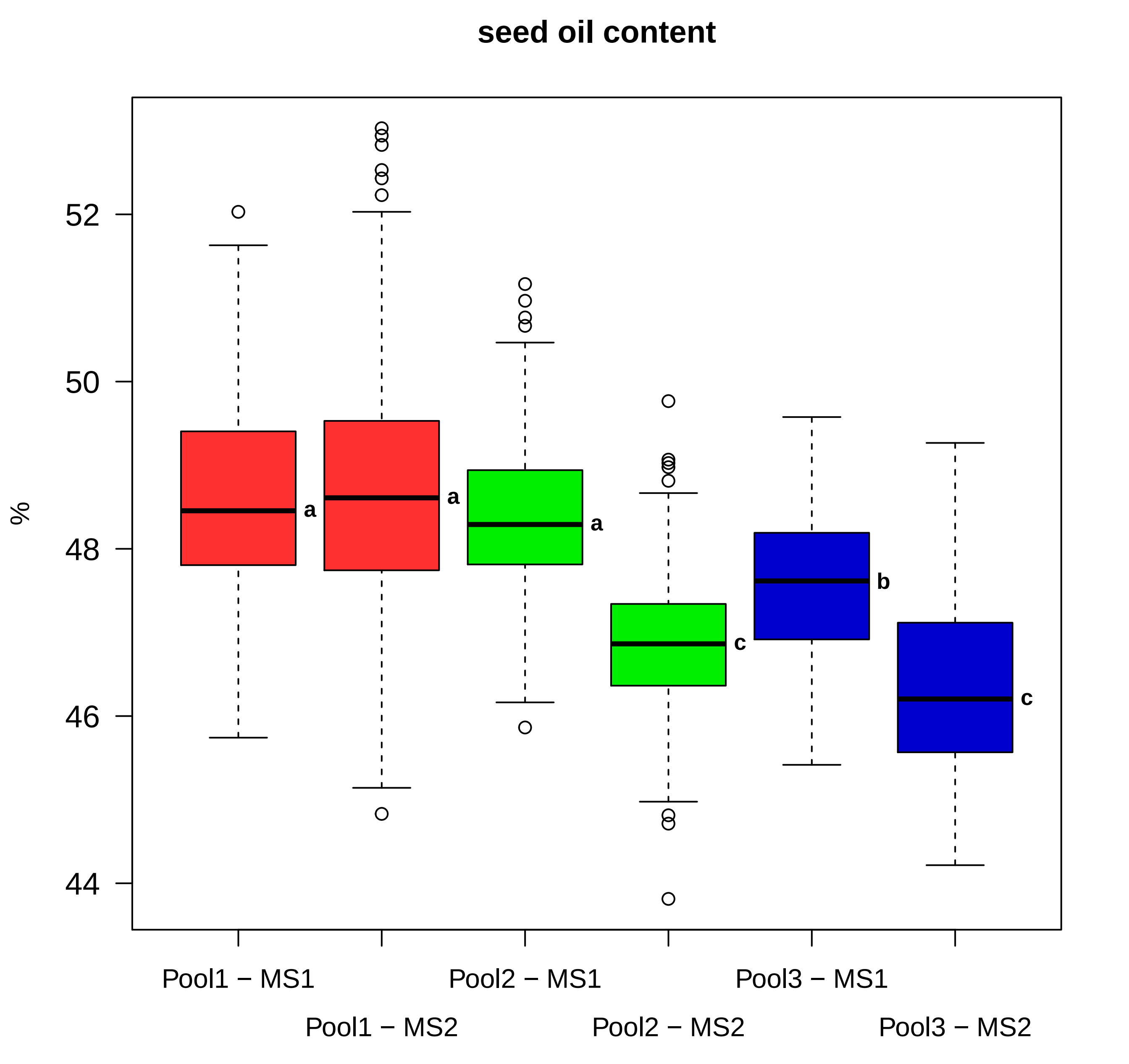

seed oil content
%
52
50
48
46
44
a
a
a
c
b
c
Pool1 − MS1
Pool1 − MS2
Pool2 − MS1
Pool2 − MS2
Pool3 − MS1
Pool3 − MS2

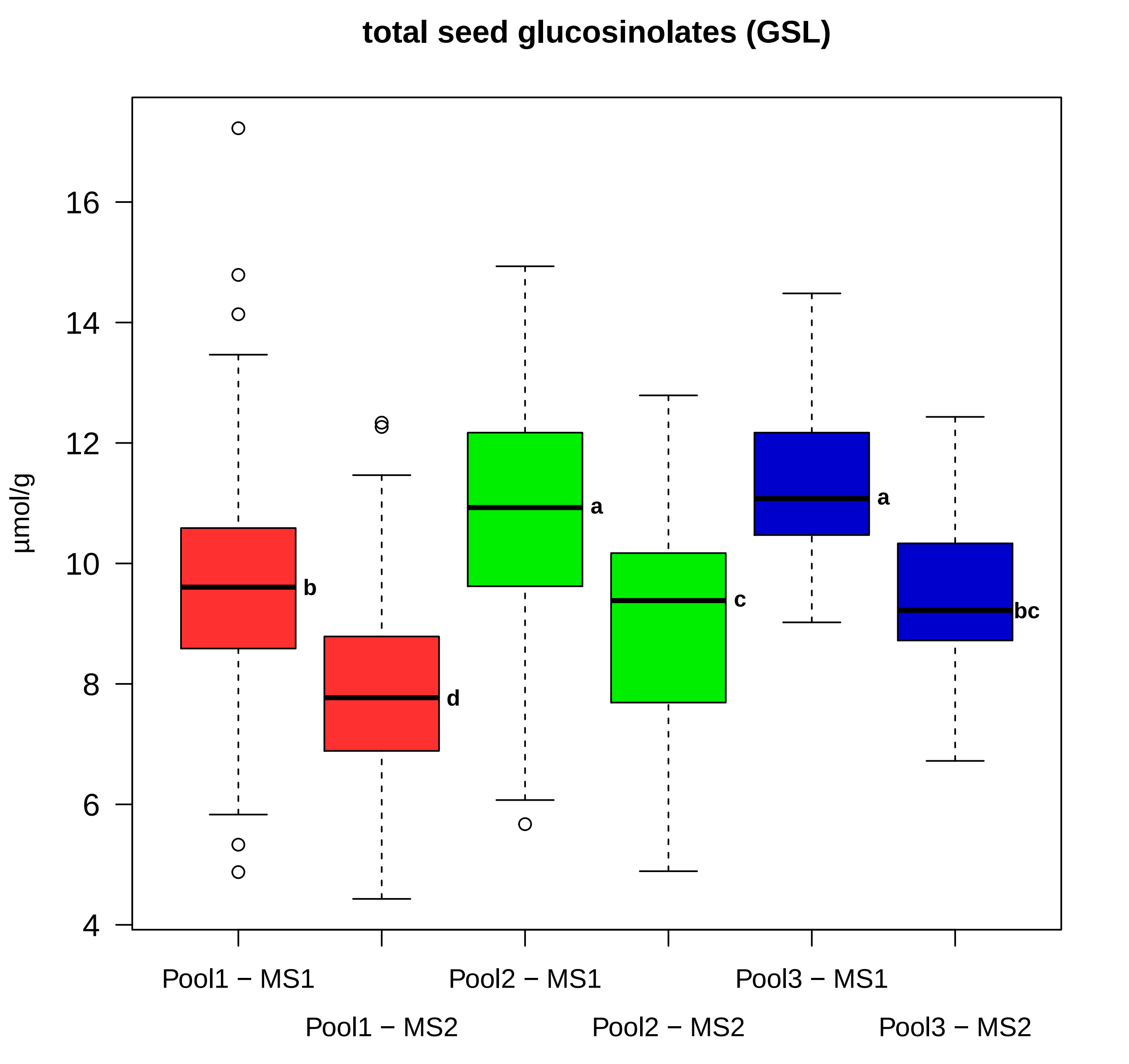

total seed glucosinolates (GSL)
µmol/g
16
14
12
10
8
6
4
b
d
a
c
a
bc
Pool1 − MS1
Pool1 − MS2
Pool2 − MS1
Pool2 − MS2
Pool3 − MS1
Pool3 − MS2

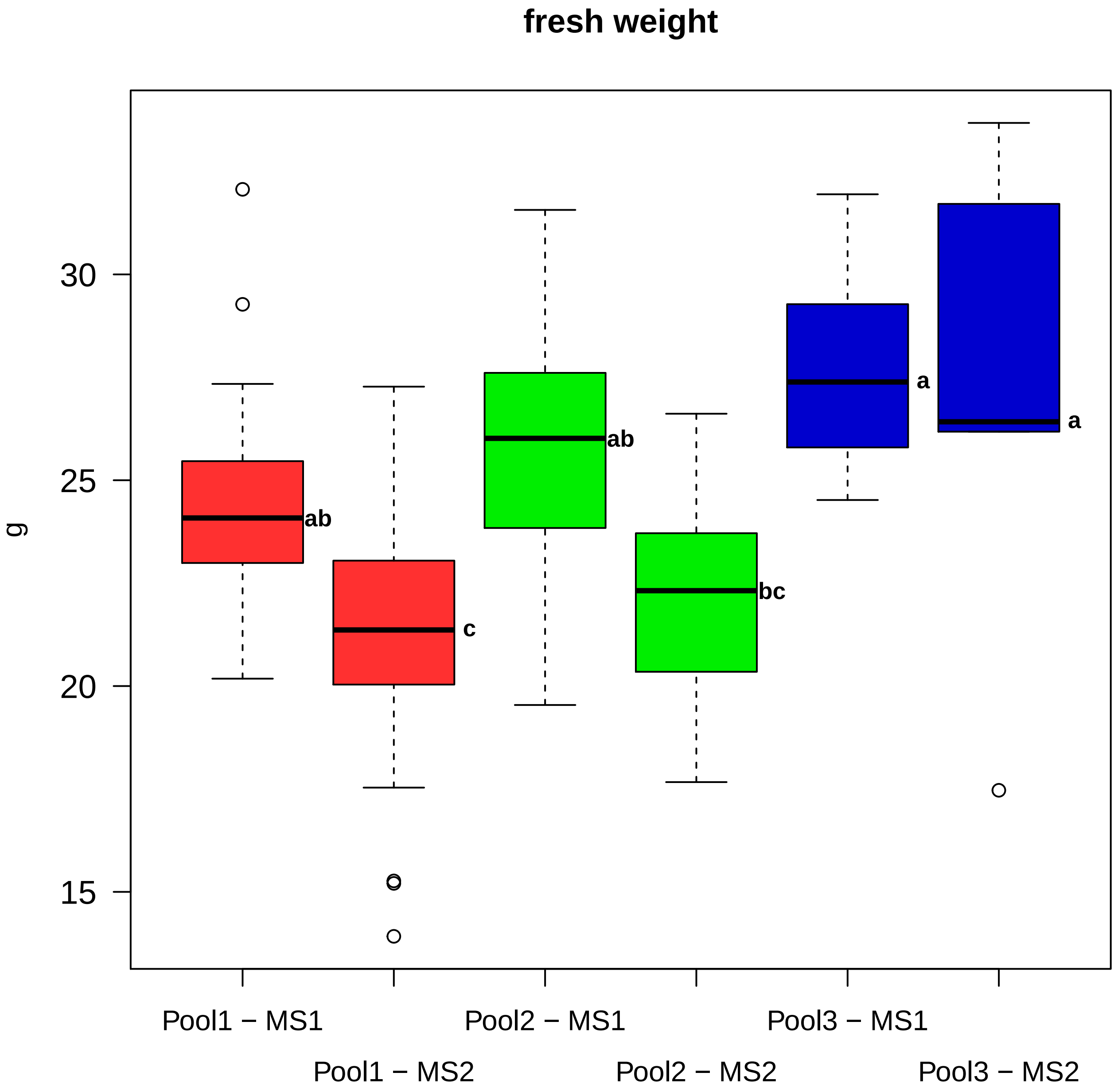

fresh weight
g
30
25
20
15
ab
c
ab
bc
a
a
Pool1 − MS1
Pool1 − MS2
Pool2 − MS1
Pool2 − MS2
Pool3 − MS1
Pool3 − MS2

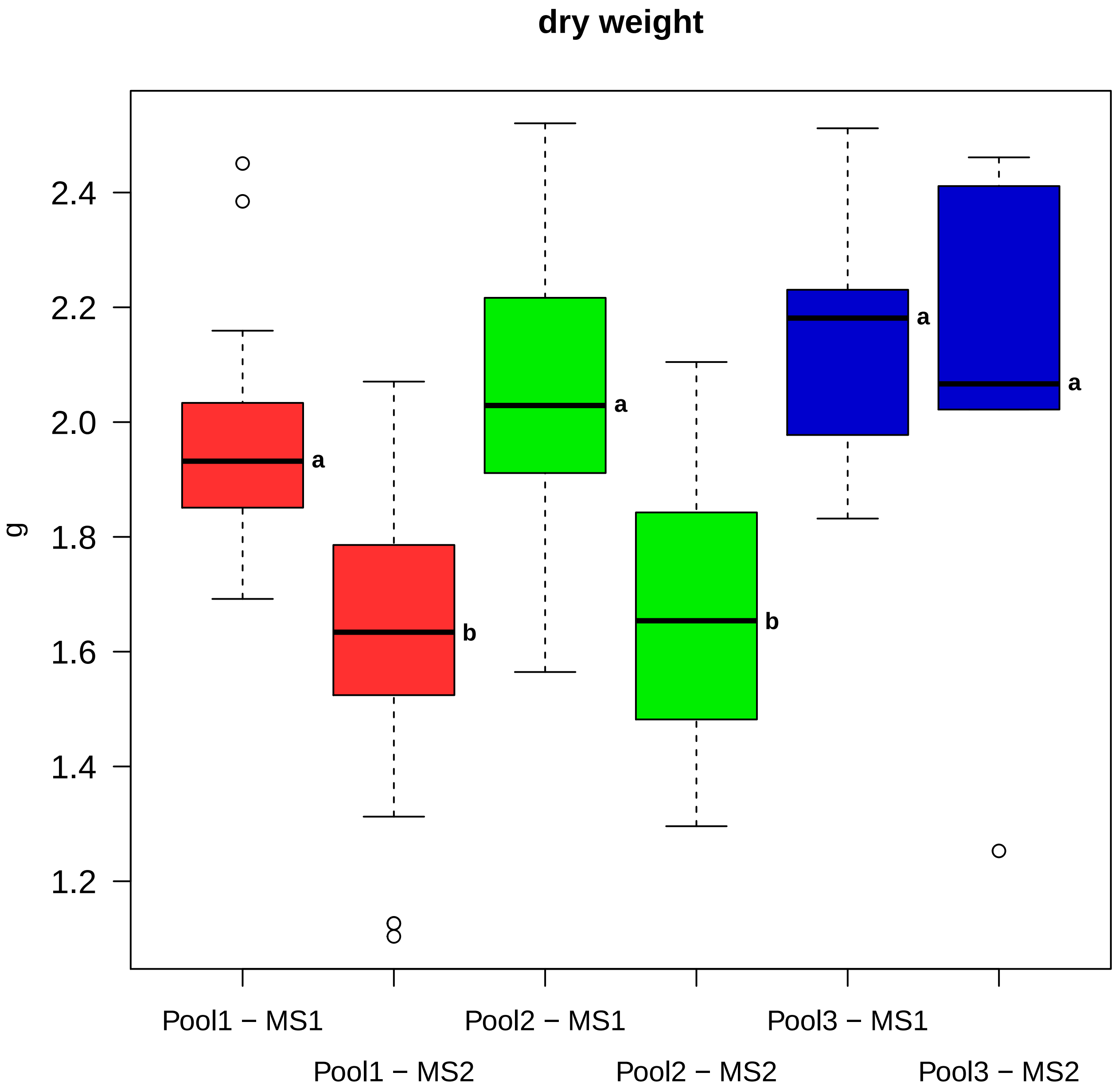

dry weight
g
2.4
2.2
2.0
1.8
1.6
1.4
1.2
a
b
a
b
a
a
Pool1 − MS1
Pool1 − MS2
Pool2 − MS1
Pool2 − MS2
Pool3 − MS1
Pool3 − MS2
